# Supplementary material for: The pulmonary mycobiome—A study of subjects with and without chronic obstructive pulmonary disease
Source: PLoS One. 2021 Apr 7;16(4):e0248967. doi: 10.1371/journal.pone.0248967 (PMC8026037; doi:10.1371/journal.pone.0248967)
Supplement: S2 Fig — Yue-Clayton measures from (A) controls and (B) participants with COPD. YC: Yue-Clayton measure. OW: oral wash, NCS: negative control sample, BAL: bronchoalveolar lavage. A Yue-Clayton measure of 0 means identical sample pairs, while a Yue-Clayton measure of 1 means unidentical sample pairs. (PDF) [file pone.0248967.s002.pdf]

The pulmonary mycobiome - a study of subjects with and without chronic obstructive pulmonary disease

Supporting Information, S2 Fig

Einar M. H. Martinsen[1*], Tomas M. L. Eagan[1,2], Elise O. Leiten[1], Ingvild Haaland[1], Gunnar R. Husebø[1,2], Kristel S. Knudsen[2], Christine Drengenes[1,2], Walter Sanseverino[3], Andreu Paytuví-Gallart[3], and Rune Nielsen[1,2]

[1]Department of Clinical Science, University of Bergen, Bergen, Norway

[2]Department of Thoracic Medicine, Haukeland University Hospital, Bergen, Norway

[3]Sequentia Biotech SL, Barcelona, Spain

* Corresponding author

E-mail: einar.martinsen@uib.no

S2 Fig. Yue-Clayton measures from (A) controls and (B) participants with COPD.

(A)

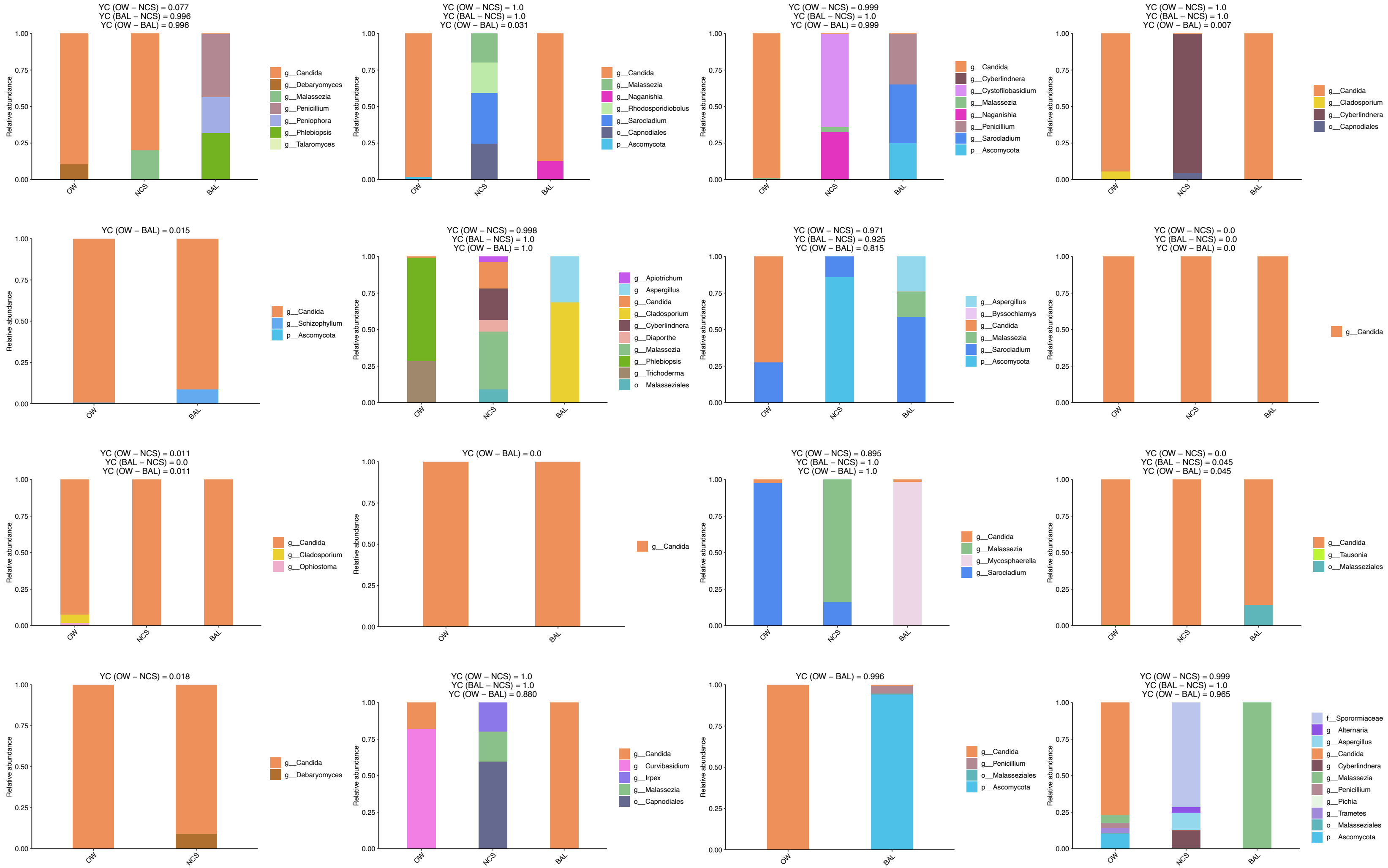

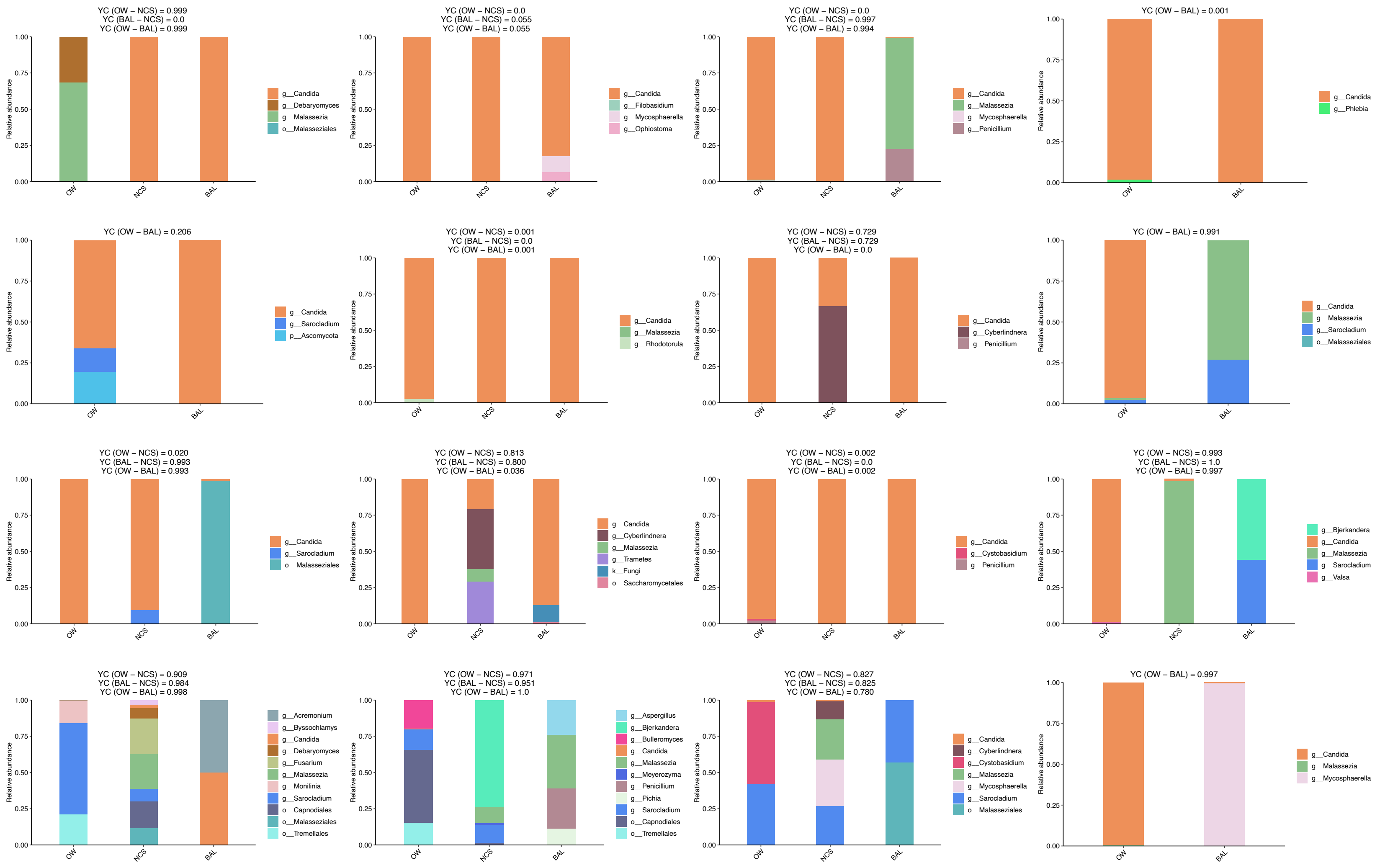
YC (OW – NCS) = 0.999
YC (BAL – NCS) = 0.0
YC (OW – BAL) = 0.999
g__Candida
g__Debaryomyces
g__Malassezia
o__Malasseziales
YC (OW – NCS) = 0.0
YC (BAL – NCS) = 0.055
YC (OW – BAL) = 0.055
g__Candida
g__Filobasidium
g__Mycosphaerella
g__Ophiostoma
YC (OW – NCS) = 0.0
YC (BAL – NCS) = 0.997
YC (OW – BAL) = 0.994
g__Candida
g__Malassezia
g__Mycosphaerella
g__Penicillium
YC (OW – BAL) = 0.001
g__Candida
g__Phlebia
YC (OW – BAL) = 0.206
g__Candida
g__Sarocladium
p__Ascomycota
YC (OW – NCS) = 0.001
YC (BAL – NCS) = 0.0
YC (OW – BAL) = 0.001
g__Candida
g__Malassezia
g__Rhodotorula
YC (OW – NCS) = 0.729
YC (BAL – NCS) = 0.729
YC (OW – BAL) = 0.0
g__Candida
g__Cyberlindnera
g__Penicillium
YC (OW – BAL) = 0.991
g__Candida
g__Malassezia
g__Sarocladium
o__Malasseziales
YC (OW – NCS) = 0.020
YC (BAL – NCS) = 0.993
YC (OW – BAL) = 0.993
g__Candida
g__Sarocladium
o__Malasseziales
YC (OW – NCS) = 0.813
YC (BAL – NCS) = 0.800
YC (OW – BAL) = 0.036
g__Candida
g__Cyberlindnera
g__Malassezia
g__Trametes
k__Fungi
o__Saccharomycetales
YC (OW – NCS) = 0.002
YC (BAL – NCS) = 0.0
YC (OW – BAL) = 0.002
g__Candida
g__Cystobasidium
g__Penicillium
YC (OW – NCS) = 0.993
YC (BAL – NCS) = 1.0
YC (OW – BAL) = 0.997
g__Bjerkandera
g__Candida
g__Malassezia
g__Sarocladium
g__Valsa
YC (OW – NCS) = 0.909
YC (BAL – NCS) = 0.984
YC (OW – BAL) = 0.998
g__Acremonium
g__Byssochlamys
g__Candida
g__Debaryomyces
g__Fusarium
g__Malassezia
g__Monilinia
g__Sarocladium
o__Capnodiales
o__Malasseziales
o__Tremellales
YC (OW – NCS) = 0.971
YC (BAL – NCS) = 0.951
YC (OW – BAL) = 1.0
g__Aspergillus
g__Bjerkandera
g__Bulleromyces
g__Candida
g__Malassezia
g__Meyerozyma
g__Penicillium
g__Pichia
g__Sarocladium
o__Capnodiales
o__Tremellales
YC (OW – NCS) = 0.827
YC (BAL – NCS) = 0.825
YC (OW – BAL) = 0.780
g__Candida
g__Cyberlindnera
g__Cystobasidium
g__Malassezia
g__Mycosphaerella
g__Sarocladium
o__Malasseziales
YC (OW – BAL) = 0.997
g__Candida
g__Malassezia
g__Mycosphaerella
Relative abundance
OW
NCS
BAL

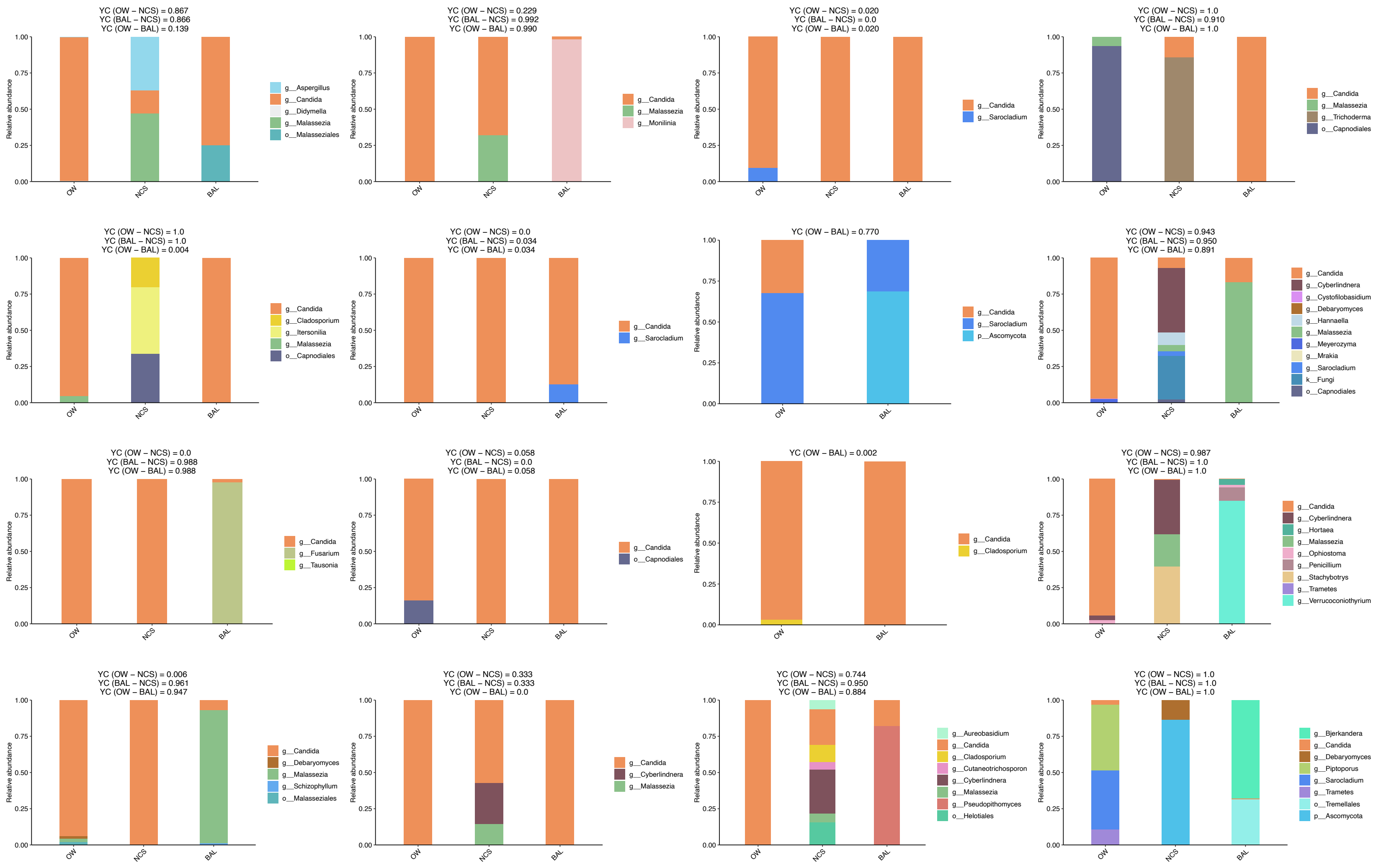
YC (OW – NCS) = 0.867
YC (BAL – NCS) = 0.866
YC (OW – BAL) = 0.139
g__Aspergillus
g__Candida
g__Didymella
g__Malassezia
o__Malasseziales
YC (OW – NCS) = 0.229
YC (BAL – NCS) = 0.992
YC (OW – BAL) = 0.990
g__Candida
g__Malassezia
g__Monilinia
YC (OW – NCS) = 0.020
YC (BAL – NCS) = 0.0
YC (OW – BAL) = 0.020
g__Candida
g__Sarocladium
YC (OW – NCS) = 1.0
YC (BAL – NCS) = 0.910
YC (OW – BAL) = 1.0
g__Candida
g__Malassezia
g__Trichoderma
o__Capnodiales
YC (OW – NCS) = 1.0
YC (BAL – NCS) = 1.0
YC (OW – BAL) = 0.004
g__Candida
g__Cladosporium
g__Itersonilia
g__Malassezia
o__Capnodiales
YC (OW – NCS) = 0.0
YC (BAL – NCS) = 0.034
YC (OW – BAL) = 0.034
g__Candida
g__Sarocladium
YC (OW – BAL) = 0.770
g__Candida
g__Sarocladium
p__Ascomycota
YC (OW – NCS) = 0.943
YC (BAL – NCS) = 0.950
YC (OW – BAL) = 0.891
g__Candida
g__Cyberlindnera
g__Cystofilobasidium
g__Debaryomyces
g__Hannaella
g__Malassezia
g__Meyerozyma
g__Mrakia
g__Sarocladium
k__Fungi
o__Capnodiales
YC (OW – NCS) = 0.0
YC (BAL – NCS) = 0.988
YC (OW – BAL) = 0.988
g__Candida
g__Fusarium
g__Tausonia
YC (OW – NCS) = 0.058
YC (BAL – NCS) = 0.0
YC (OW – BAL) = 0.058
g__Candida
o__Capnodiales
YC (OW – BAL) = 0.002
g__Candida
g__Cladosporium
YC (OW – NCS) = 0.987
YC (BAL – NCS) = 1.0
YC (OW – BAL) = 1.0
g__Candida
g__Cyberlindnera
g__Hortaea
g__Malassezia
g__Ophiostoma
g__Penicillium
g__Stachybotrys
g__Trametes
g__Verrucoconiothyrium
YC (OW – NCS) = 0.006
YC (BAL – NCS) = 0.961
YC (OW – BAL) = 0.947
g__Candida
g__Debaryomyces
g__Malassezia
g__Schizophyllum
o__Malasseziales
YC (OW – NCS) = 0.333
YC (BAL – NCS) = 0.333
YC (OW – BAL) = 0.0
g__Candida
g__Cyberlindnera
g__Malassezia
YC (OW – NCS) = 0.744
YC (BAL – NCS) = 0.950
YC (OW – BAL) = 0.884
g__Aureobasidium
g__Candida
g__Cladosporium
g__Cutaneotrichosporon
g__Cyberlindnera
g__Malassezia
g__Pseudopithomyces
o__Helotiales
YC (OW – NCS) = 1.0
YC (BAL – NCS) = 1.0
YC (OW – BAL) = 1.0
g__Bjerkandera
g__Candida
g__Debaryomyces
g__Piptoporus
g__Sarocladium
g__Trametes
o__Tremellales
p__Ascomycota
Relative abundance
OW
NCS
BAL

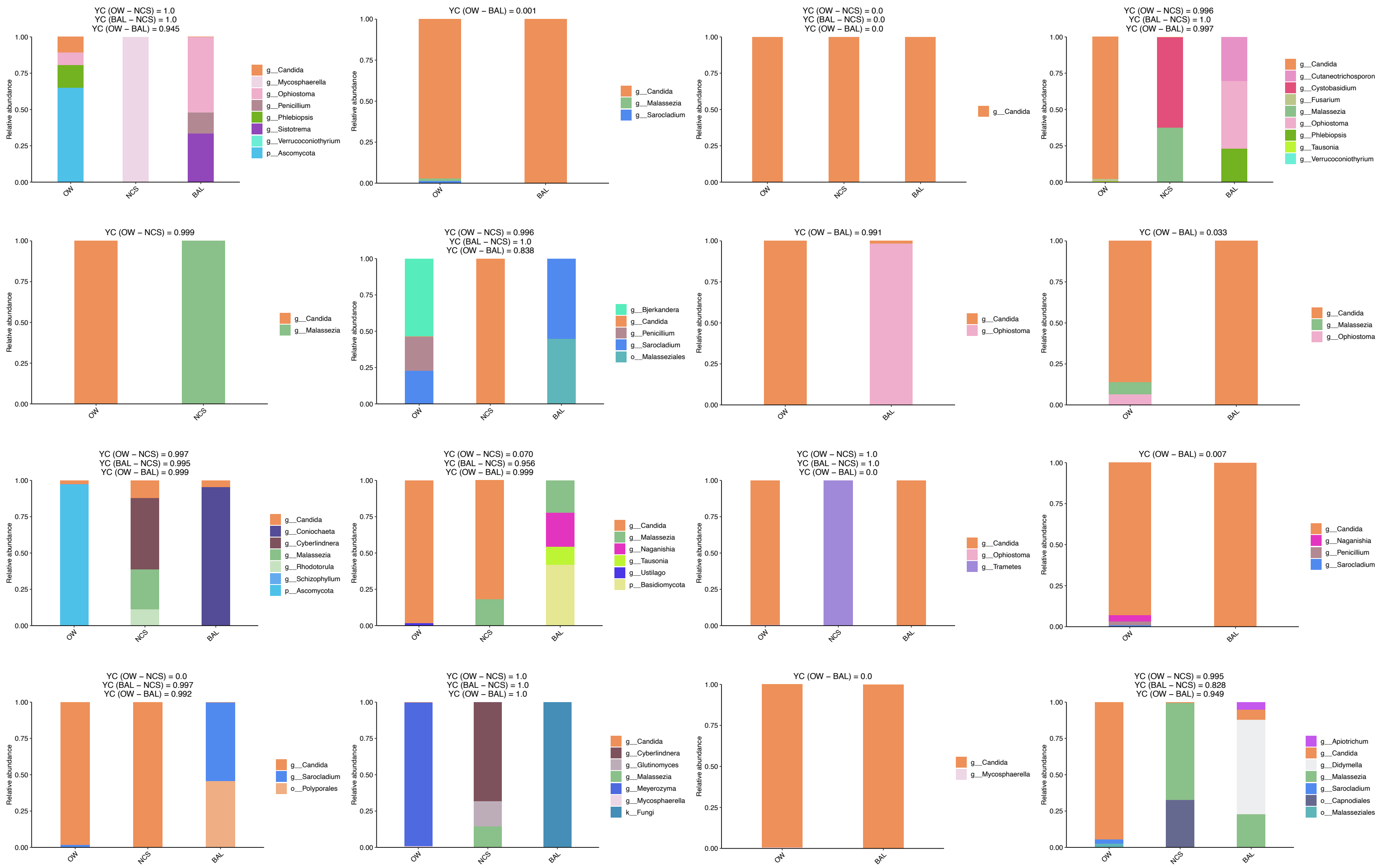

YC (OW – NCS) = 1.0
YC (BAL – NCS) = 1.0
YC (OW – BAL) = 0.945
Relative abundance
OW
NCS
BAL
g__Candida
g__Mycosphaerella
g__Ophiostoma
g__Penicillium
g__Phlebiopsis
g__Sistotrema
g__Verrucoconiothyrium
p__Ascomycota
YC (OW – BAL) = 0.001
g__Candida
g__Malassezia
g__Sarocladium
YC (OW – NCS) = 0.0
YC (BAL – NCS) = 0.0
YC (OW – BAL) = 0.0
g__Candida
YC (OW – NCS) = 0.996
YC (BAL – NCS) = 1.0
YC (OW – BAL) = 0.997
g__Candida
g__Cutaneotrichosporon
g__Cystobasidium
g__Fusarium
g__Malassezia
g__Ophiostoma
g__Phlebiopsis
g__Tausonia
g__Verrucoconiothyrium
YC (OW – NCS) = 0.999
g__Candida
g__Malassezia
YC (OW – NCS) = 0.996
YC (BAL – NCS) = 1.0
YC (OW – BAL) = 0.838
g__Bjerkandera
g__Candida
g__Penicillium
g__Sarocladium
o__Malasseziales
YC (OW – BAL) = 0.991
g__Candida
g__Ophiostoma
YC (OW – BAL) = 0.033
g__Candida
g__Malassezia
g__Ophiostoma
YC (OW – NCS) = 0.997
YC (BAL – NCS) = 0.995
YC (OW – BAL) = 0.999
g__Candida
g__Coniochaeta
g__Cyberlindnera
g__Malassezia
g__Rhodotorula
g__Schizophyllum
p__Ascomycota
YC (OW – NCS) = 0.070
YC (BAL – NCS) = 0.956
YC (OW – BAL) = 0.999
g__Candida
g__Malassezia
g__Naganishia
g__Tausonia
g__Ustilago
p__Basidiomycota
YC (OW – NCS) = 1.0
YC (BAL – NCS) = 1.0
YC (OW – BAL) = 0.0
g__Candida
g__Ophiostoma
g__Trametes
YC (OW – BAL) = 0.007
g__Candida
g__Naganishia
g__Penicillium
g__Sarocladium
YC (OW – NCS) = 0.0
YC (BAL – NCS) = 0.997
YC (OW – BAL) = 0.992
g__Candida
g__Sarocladium
o__Polyporales
YC (OW – NCS) = 1.0
YC (BAL – NCS) = 1.0
YC (OW – BAL) = 1.0
g__Candida
g__Cyberlindnera
g__Glutinomyces
g__Malassezia
g__Meyerozyma
g__Mycosphaerella
k__Fungi
YC (OW – BAL) = 0.0
g__Candida
g__Mycosphaerella
YC (OW – NCS) = 0.995
YC (BAL – NCS) = 0.828
YC (OW – BAL) = 0.949
g__Apiotrichum
g__Candida
g__Didymella
g__Malassezia
g__Sarocladium
o__Capnodiales
o__Malasseziales

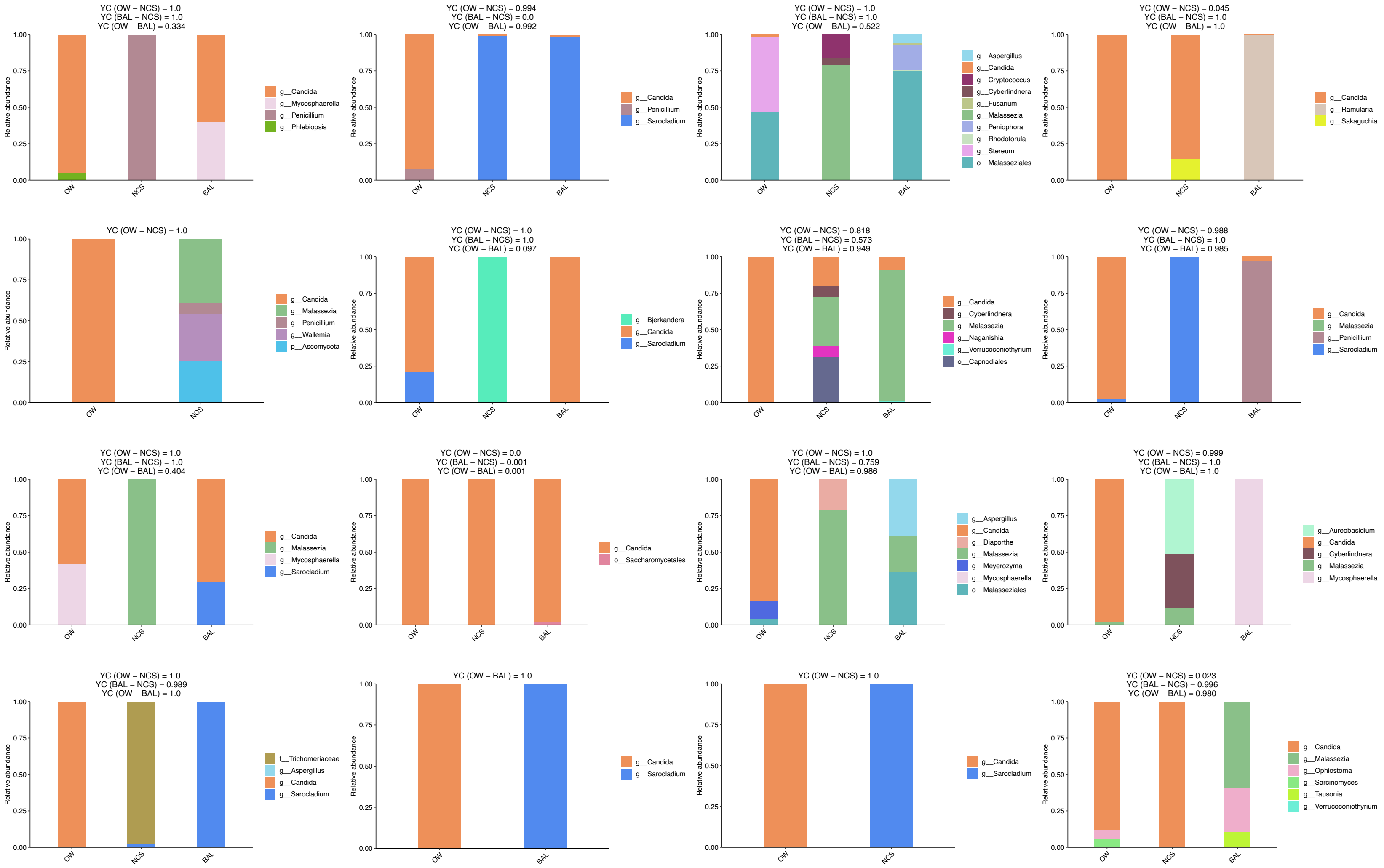
YC (OW – NCS) = 1.0
YC (BAL – NCS) = 1.0
YC (OW – BAL) = 0.334
g__Candida
g__Mycosphaerella
g__Penicillium
g__Phlebiopsis
YC (OW – NCS) = 0.994
YC (BAL – NCS) = 0.0
YC (OW – BAL) = 0.992
g__Candida
g__Penicillium
g__Sarocladium
YC (OW – NCS) = 1.0
YC (BAL – NCS) = 1.0
YC (OW – BAL) = 0.522
g__Aspergillus
g__Candida
g__Cryptococcus
g__Cyberlindnera
g__Fusarium
g__Malassezia
g__Peniophora
g__Rhodotorula
g__Stereum
o__Malasseziales
YC (OW – NCS) = 0.045
YC (BAL – NCS) = 1.0
YC (OW – BAL) = 1.0
g__Candida
g__Ramularia
g__Sakaguchia
YC (OW – NCS) = 1.0
g__Candida
g__Malassezia
g__Penicillium
g__Wallemia
p__Ascomycota
YC (OW – NCS) = 1.0
YC (BAL – NCS) = 1.0
YC (OW – BAL) = 0.097
g__Bjerkandera
g__Candida
g__Sarocladium
YC (OW – NCS) = 0.818
YC (BAL – NCS) = 0.573
YC (OW – BAL) = 0.949
g__Candida
g__Cyberlindnera
g__Malassezia
g__Naganishia
g__Verrucoconiothyrium
o__Capnodiales
YC (OW – NCS) = 0.988
YC (BAL – NCS) = 1.0
YC (OW – BAL) = 0.985
g__Candida
g__Malassezia
g__Penicillium
g__Sarocladium
YC (OW – NCS) = 1.0
YC (BAL – NCS) = 1.0
YC (OW – BAL) = 0.404
g__Candida
g__Malassezia
g__Mycosphaerella
g__Sarocladium
YC (OW – NCS) = 0.0
YC (BAL – NCS) = 0.001
YC (OW – BAL) = 0.001
g__Candida
o__Saccharomycetales
YC (OW – NCS) = 1.0
YC (BAL – NCS) = 0.759
YC (OW – BAL) = 0.986
g__Aspergillus
g__Candida
g__Diaporthe
g__Malassezia
g__Meyerozyma
g__Mycosphaerella
o__Malasseziales
YC (OW – NCS) = 0.999
YC (BAL – NCS) = 1.0
YC (OW – BAL) = 1.0
g__Aureobasidium
g__Candida
g__Cyberlindnera
g__Malassezia
g__Mycosphaerella
YC (OW – NCS) = 1.0
YC (BAL – NCS) = 0.989
YC (OW – BAL) = 1.0
f__Trichomeriaceae
g__Aspergillus
g__Candida
g__Sarocladium
YC (OW – BAL) = 1.0
g__Candida
g__Sarocladium
YC (OW – NCS) = 1.0
g__Candida
g__Sarocladium
YC (OW – NCS) = 0.023
YC (BAL – NCS) = 0.996
YC (OW – BAL) = 0.980
g__Candida
g__Malassezia
g__Ophiostoma
g__Sarcinomyces
g__Tausonia
g__Verrucoconiothyrium
Relative abundance
OW
NCS
BAL

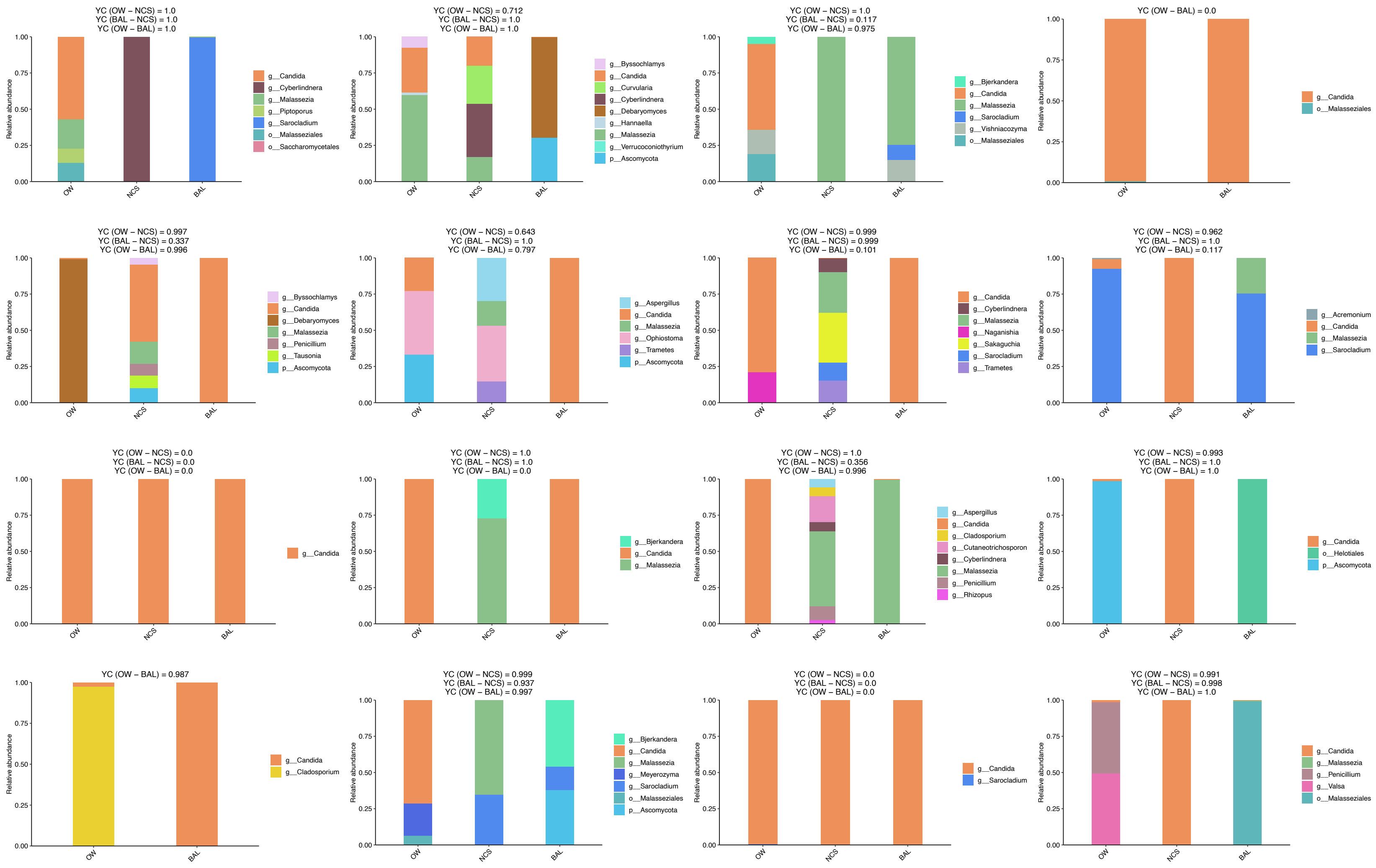
YC (OW – NCS) = 1.0
YC (BAL – NCS) = 1.0
YC (OW – BAL) = 1.0
g__Candida
g__Cyberlindnera
g__Malassezia
g__Piptoporus
g__Sarocladium
o__Malasseziales
o__Saccharomycetales
YC (OW – NCS) = 0.712
YC (BAL – NCS) = 1.0
YC (OW – BAL) = 1.0
g__Byssochlamys
g__Candida
g__Curvularia
g__Cyberlindnera
g__Debaryomyces
g__Hannaella
g__Malassezia
g__Verrucoconiothyrium
p__Ascomycota
YC (OW – NCS) = 1.0
YC (BAL – NCS) = 0.117
YC (OW – BAL) = 0.975
g__Bjerkandera
g__Candida
g__Malassezia
g__Sarocladium
g__Vishniacozyma
o__Malasseziales
YC (OW – BAL) = 0.0
g__Candida
o__Malasseziales
YC (OW – NCS) = 0.997
YC (BAL – NCS) = 0.337
YC (OW – BAL) = 0.996
g__Byssochlamys
g__Candida
g__Debaryomyces
g__Malassezia
g__Penicillium
g__Tausonia
p__Ascomycota
YC (OW – NCS) = 0.643
YC (BAL – NCS) = 1.0
YC (OW – BAL) = 0.797
g__Aspergillus
g__Candida
g__Malassezia
g__Ophiostoma
g__Trametes
p__Ascomycota
YC (OW – NCS) = 0.999
YC (BAL – NCS) = 0.999
YC (OW – BAL) = 0.101
g__Candida
g__Cyberlindnera
g__Malassezia
g__Naganishia
g__Sakaguchia
g__Sarocladium
g__Trametes
YC (OW – NCS) = 0.962
YC (BAL – NCS) = 1.0
YC (OW – BAL) = 0.117
g__Acremonium
g__Candida
g__Malassezia
g__Sarocladium
YC (OW – NCS) = 0.0
YC (BAL – NCS) = 0.0
YC (OW – BAL) = 0.0
g__Candida
YC (OW – NCS) = 1.0
YC (BAL – NCS) = 1.0
YC (OW – BAL) = 0.0
g__Bjerkandera
g__Candida
g__Malassezia
YC (OW – NCS) = 1.0
YC (BAL – NCS) = 0.356
YC (OW – BAL) = 0.996
g__Aspergillus
g__Candida
g__Cladosporium
g__Cutaneotrichosporon
g__Cyberlindnera
g__Malassezia
g__Penicillium
g__Rhizopus
YC (OW – NCS) = 0.993
YC (BAL – NCS) = 1.0
YC (OW – BAL) = 1.0
g__Candida
o__Helotiales
p__Ascomycota
YC (OW – BAL) = 0.987
g__Candida
g__Cladosporium
YC (OW – NCS) = 0.999
YC (BAL – NCS) = 0.937
YC (OW – BAL) = 0.997
g__Bjerkandera
g__Candida
g__Malassezia
g__Meyerozyma
g__Sarocladium
o__Malasseziales
p__Ascomycota
YC (OW – NCS) = 0.0
YC (BAL – NCS) = 0.0
YC (OW – BAL) = 0.0
g__Candida
g__Sarocladium
YC (OW – NCS) = 0.991
YC (BAL – NCS) = 0.998
YC (OW – BAL) = 1.0
g__Candida
g__Malassezia
g__Penicillium
g__Valsa
o__Malasseziales
Relative abundance
OW
NCS
BAL

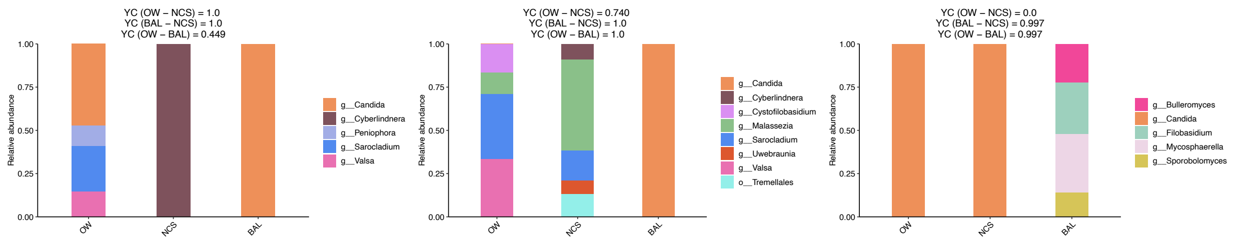
YC (OW – NCS) = 1.0
YC (BAL – NCS) = 1.0
YC (OW – BAL) = 0.449
Relative abundance
1.00
0.75
0.50
0.25
0.00
OW
NCS
BAL
g__Candida
g__Cyberlindnera
g__Peniophora
g__Sarocladium
g__Valsa
YC (OW – NCS) = 0.740
YC (BAL – NCS) = 1.0
YC (OW – BAL) = 1.0
g__Candida
g__Cyberlindnera
g__Cystofilobasidium
g__Malassezia
g__Sarocladium
g__Uwebraunia
g__Valsa
o__Tremellales
YC (OW – NCS) = 0.0
YC (BAL – NCS) = 0.997
YC (OW – BAL) = 0.997
g__Bulleromyces
g__Candida
g__Filobasidium
g__Mycosphaerella
g__Sporobolomyces

(B)

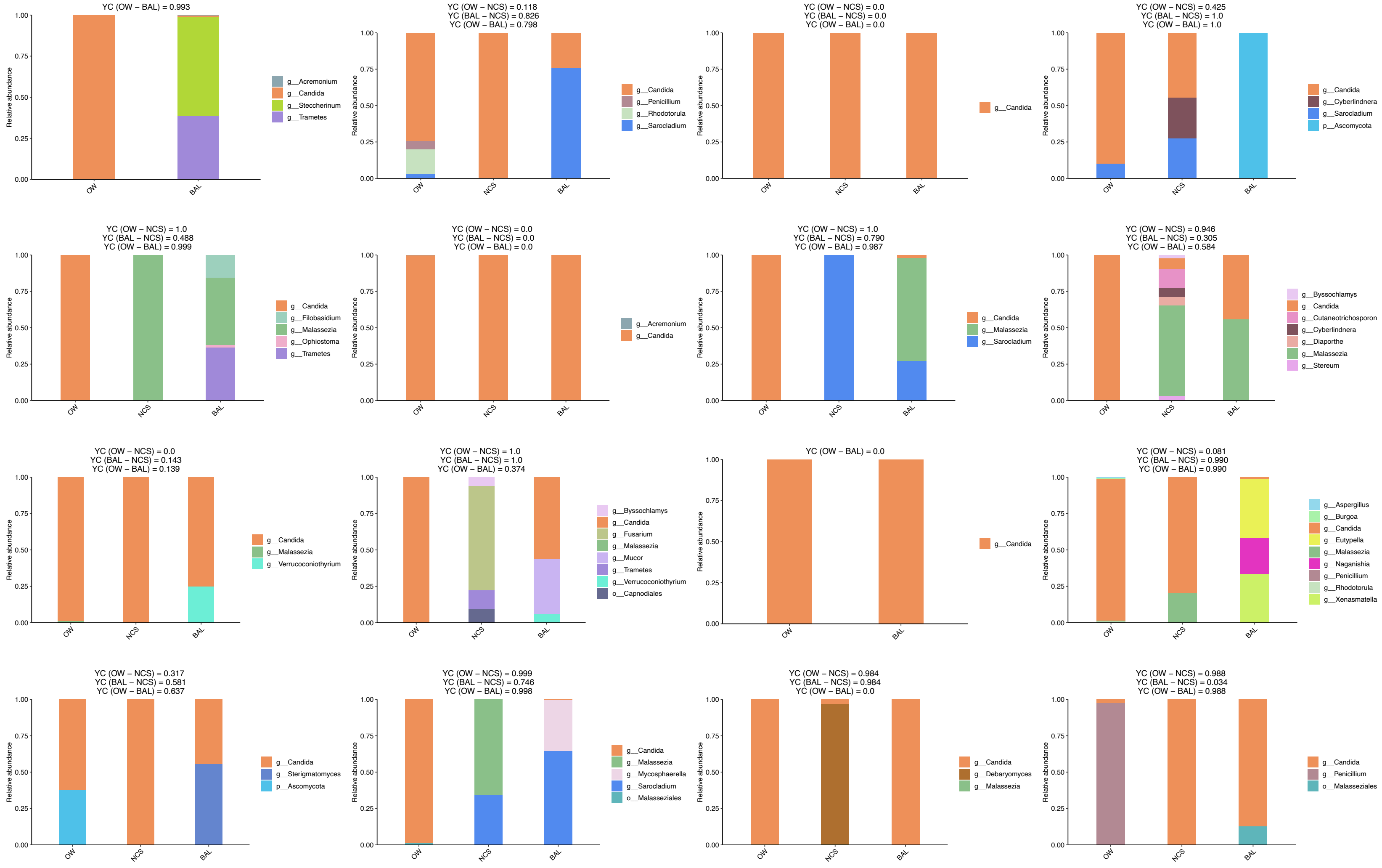
YC (OW – BAL) = 0.993
Relative abundance
OW
BAL
g__Acremonium
g__Candida
g__Steccherinum
g__Trametes
YC (OW – NCS) = 0.118
YC (BAL – NCS) = 0.826
YC (OW – BAL) = 0.798
NCS
g__Penicillium
g__Rhodotorula
g__Sarocladium
YC (OW – NCS) = 0.0
YC (BAL – NCS) = 0.0
YC (OW – BAL) = 0.0
YC (OW – NCS) = 0.425
YC (BAL – NCS) = 1.0
YC (OW – BAL) = 1.0
g__Cyberlindnera
p__Ascomycota
YC (OW – NCS) = 1.0
YC (BAL – NCS) = 0.488
YC (OW – BAL) = 0.999
g__Filobasidium
g__Malassezia
g__Ophiostoma
YC (OW – NCS) = 1.0
YC (BAL – NCS) = 0.790
YC (OW – BAL) = 0.987
YC (OW – NCS) = 0.946
YC (BAL – NCS) = 0.305
YC (OW – BAL) = 0.584
g__Byssochlamys
g__Cutaneotrichosporon
g__Diaporthe
g__Stereum
YC (OW – NCS) = 0.0
YC (BAL – NCS) = 0.143
YC (OW – BAL) = 0.139
g__Verrucoconiothyrium
YC (OW – NCS) = 1.0
YC (BAL – NCS) = 1.0
YC (OW – BAL) = 0.374
g__Fusarium
g__Mucor
o__Capnodiales
YC (OW – BAL) = 0.0
YC (OW – NCS) = 0.081
YC (BAL – NCS) = 0.990
YC (OW – BAL) = 0.990
g__Aspergillus
g__Burgoa
g__Eutypella
g__Naganishia
g__Xenasmatella
YC (OW – NCS) = 0.317
YC (BAL – NCS) = 0.581
YC (OW – BAL) = 0.637
g__Sterigmatomyces
YC (OW – NCS) = 0.999
YC (BAL – NCS) = 0.746
YC (OW – BAL) = 0.998
g__Mycosphaerella
o__Malasseziales
YC (OW – NCS) = 0.984
YC (BAL – NCS) = 0.984
YC (OW – BAL) = 0.0
g__Debaryomyces
YC (OW – NCS) = 0.988
YC (BAL – NCS) = 0.034
YC (OW – BAL) = 0.988

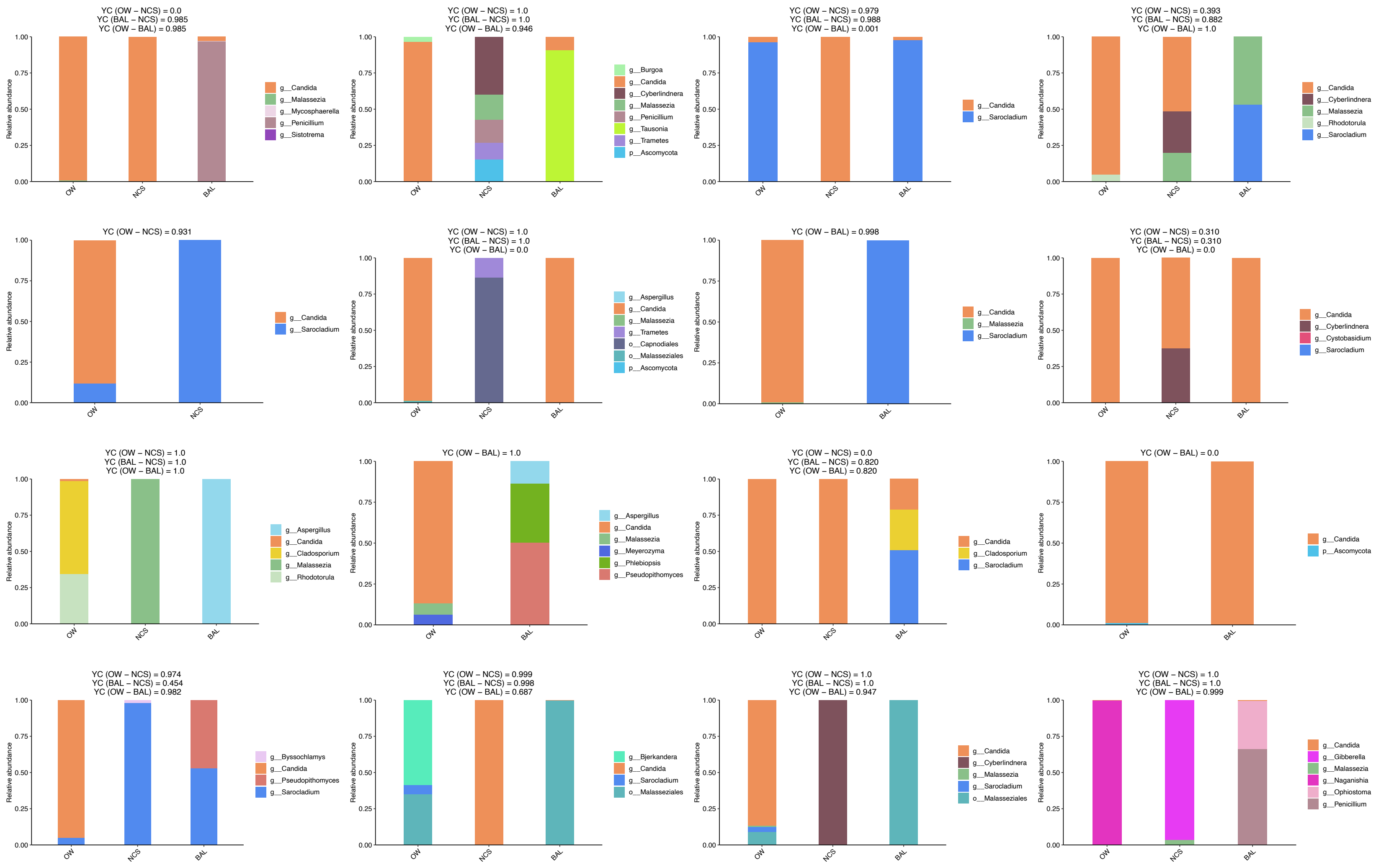
YC (OW – NCS) = 0.0
YC (BAL – NCS) = 0.985
YC (OW – BAL) = 0.985
Relative abundance
1.00
0.75
0.50
0.25
0.00
OW
NCS
BAL
g__Candida
g__Malassezia
g__Mycosphaerella
g__Penicillium
g__Sistotrema
YC (OW – NCS) = 1.0
YC (BAL – NCS) = 1.0
YC (OW – BAL) = 0.946
g__Burgoa
g__Candida
g__Cyberlindnera
g__Malassezia
g__Penicillium
g__Tausonia
g__Trametes
p__Ascomycota
YC (OW – NCS) = 0.979
YC (BAL – NCS) = 0.988
YC (OW – BAL) = 0.001
g__Candida
g__Sarocladium
YC (OW – NCS) = 0.393
YC (BAL – NCS) = 0.882
YC (OW – BAL) = 1.0
g__Candida
g__Cyberlindnera
g__Malassezia
g__Rhodotorula
g__Sarocladium
YC (OW – NCS) = 0.931
g__Candida
g__Sarocladium
YC (OW – NCS) = 1.0
YC (BAL – NCS) = 1.0
YC (OW – BAL) = 0.0
g__Aspergillus
g__Candida
g__Malassezia
g__Trametes
o__Capnodiales
o__Malasseziales
p__Ascomycota
YC (OW – BAL) = 0.998
g__Candida
g__Malassezia
g__Sarocladium
YC (OW – NCS) = 0.310
YC (BAL – NCS) = 0.310
YC (OW – BAL) = 0.0
g__Candida
g__Cyberlindnera
g__Cystobasidium
g__Sarocladium
YC (OW – NCS) = 1.0
YC (BAL – NCS) = 1.0
YC (OW – BAL) = 1.0
g__Aspergillus
g__Candida
g__Cladosporium
g__Malassezia
g__Rhodotorula
YC (OW – BAL) = 1.0
g__Aspergillus
g__Candida
g__Malassezia
g__Meyerozyma
g__Phlebiopsis
g__Pseudopithomyces
YC (OW – NCS) = 0.0
YC (BAL – NCS) = 0.820
YC (OW – BAL) = 0.820
g__Candida
g__Cladosporium
g__Sarocladium
YC (OW – BAL) = 0.0
g__Candida
p__Ascomycota
YC (OW – NCS) = 0.974
YC (BAL – NCS) = 0.454
YC (OW – BAL) = 0.982
g__Byssochlamys
g__Candida
g__Pseudopithomyces
g__Sarocladium
YC (OW – NCS) = 0.999
YC (BAL – NCS) = 0.998
YC (OW – BAL) = 0.687
g__Bjerkandera
g__Candida
g__Sarocladium
o__Malasseziales
YC (OW – NCS) = 1.0
YC (BAL – NCS) = 1.0
YC (OW – BAL) = 0.947
g__Candida
g__Cyberlindnera
g__Malassezia
g__Sarocladium
o__Malasseziales
YC (OW – NCS) = 1.0
YC (BAL – NCS) = 1.0
YC (OW – BAL) = 0.999
g__Candida
g__Gibberella
g__Malassezia
g__Naganishia
g__Ophiostoma
g__Penicillium

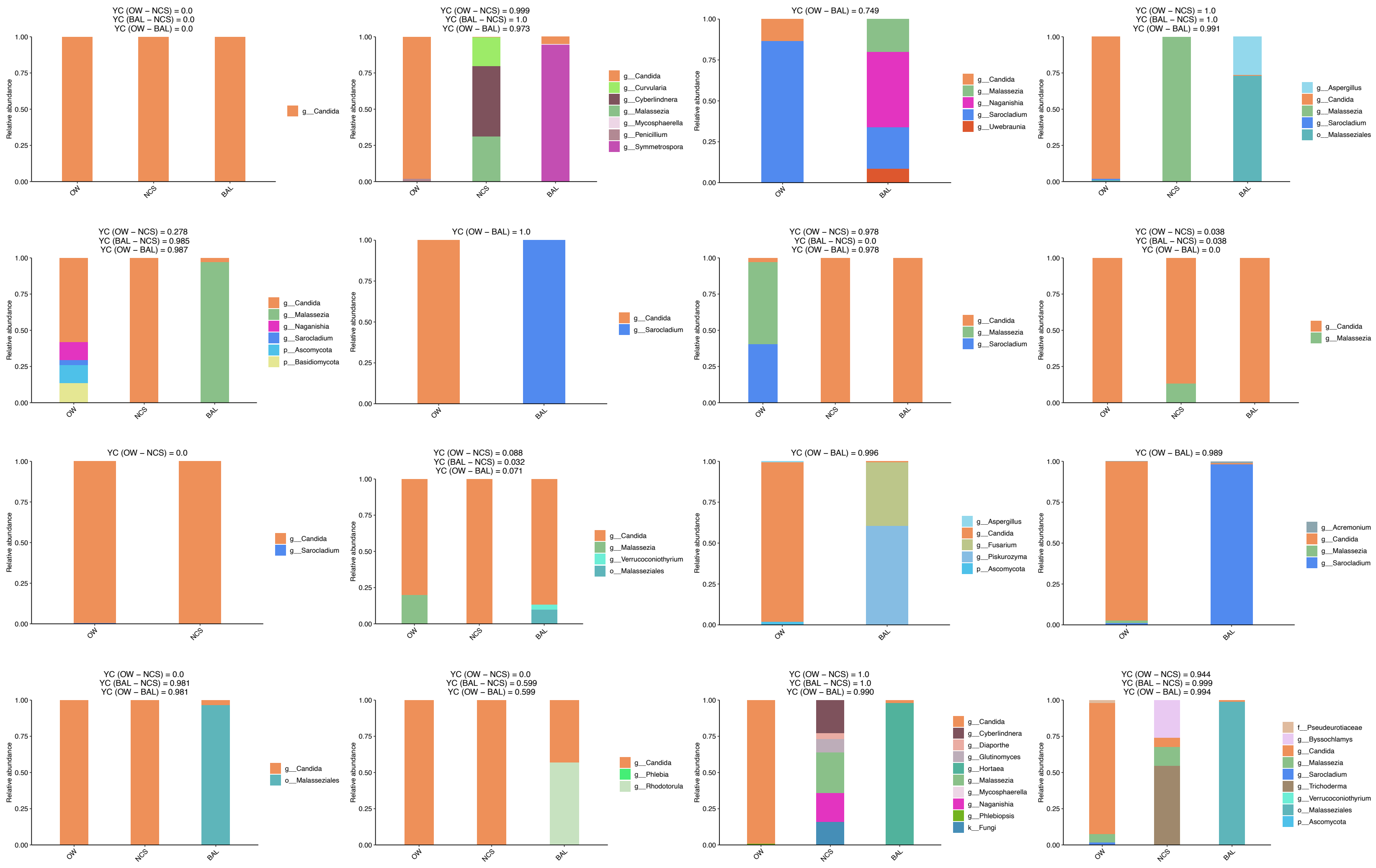

YC (OW – NCS) = 0.0
YC (BAL – NCS) = 0.0
YC (OW – BAL) = 0.0
g__Candida
YC (OW – NCS) = 0.999
YC (BAL – NCS) = 1.0
YC (OW – BAL) = 0.973
g__Candida
g__Curvularia
g__Cyberlindnera
g__Malassezia
g__Mycosphaerella
g__Penicillium
g__Symmetrospora
YC (OW – BAL) = 0.749
g__Candida
g__Malassezia
g__Naganishia
g__Sarocladium
g__Uwebraunia
YC (OW – NCS) = 1.0
YC (BAL – NCS) = 1.0
YC (OW – BAL) = 0.991
g__Aspergillus
g__Candida
g__Malassezia
g__Sarocladium
o__Malasseziales
YC (OW – NCS) = 0.278
YC (BAL – NCS) = 0.985
YC (OW – BAL) = 0.987
g__Candida
g__Malassezia
g__Naganishia
g__Sarocladium
p__Ascomycota
p__Basidiomycota
YC (OW – BAL) = 1.0
g__Candida
g__Sarocladium
YC (OW – NCS) = 0.978
YC (BAL – NCS) = 0.0
YC (OW – BAL) = 0.978
g__Candida
g__Malassezia
g__Sarocladium
YC (OW – NCS) = 0.038
YC (BAL – NCS) = 0.038
YC (OW – BAL) = 0.0
g__Candida
g__Malassezia
YC (OW – NCS) = 0.0
g__Candida
g__Sarocladium
YC (OW – NCS) = 0.088
YC (BAL – NCS) = 0.032
YC (OW – BAL) = 0.071
g__Candida
g__Malassezia
g__Verrucoconiothyrium
o__Malasseziales
YC (OW – BAL) = 0.996
g__Aspergillus
g__Candida
g__Fusarium
g__Piskurozyma
p__Ascomycota
YC (OW – BAL) = 0.989
g__Acremonium
g__Candida
g__Malassezia
g__Sarocladium
YC (OW – NCS) = 0.0
YC (BAL – NCS) = 0.981
YC (OW – BAL) = 0.981
g__Candida
o__Malasseziales
YC (OW – NCS) = 0.0
YC (BAL – NCS) = 0.599
YC (OW – BAL) = 0.599
g__Candida
g__Phlebia
g__Rhodotorula
YC (OW – NCS) = 1.0
YC (BAL – NCS) = 1.0
YC (OW – BAL) = 0.990
g__Candida
g__Cyberlindnera
g__Diaporthe
g__Glutinomyces
g__Hortaea
g__Malassezia
g__Mycosphaerella
g__Naganishia
g__Phlebiopsis
k__Fungi
YC (OW – NCS) = 0.944
YC (BAL – NCS) = 0.999
YC (OW – BAL) = 0.994
f__Pseudeurotiaceae
g__Byssochlamys
g__Candida
g__Malassezia
g__Sarocladium
g__Trichoderma
g__Verrucoconiothyrium
o__Malasseziales
p__Ascomycota
Relative abundance
0.00
0.25
0.50
0.75
1.00
OW
NCS
BAL

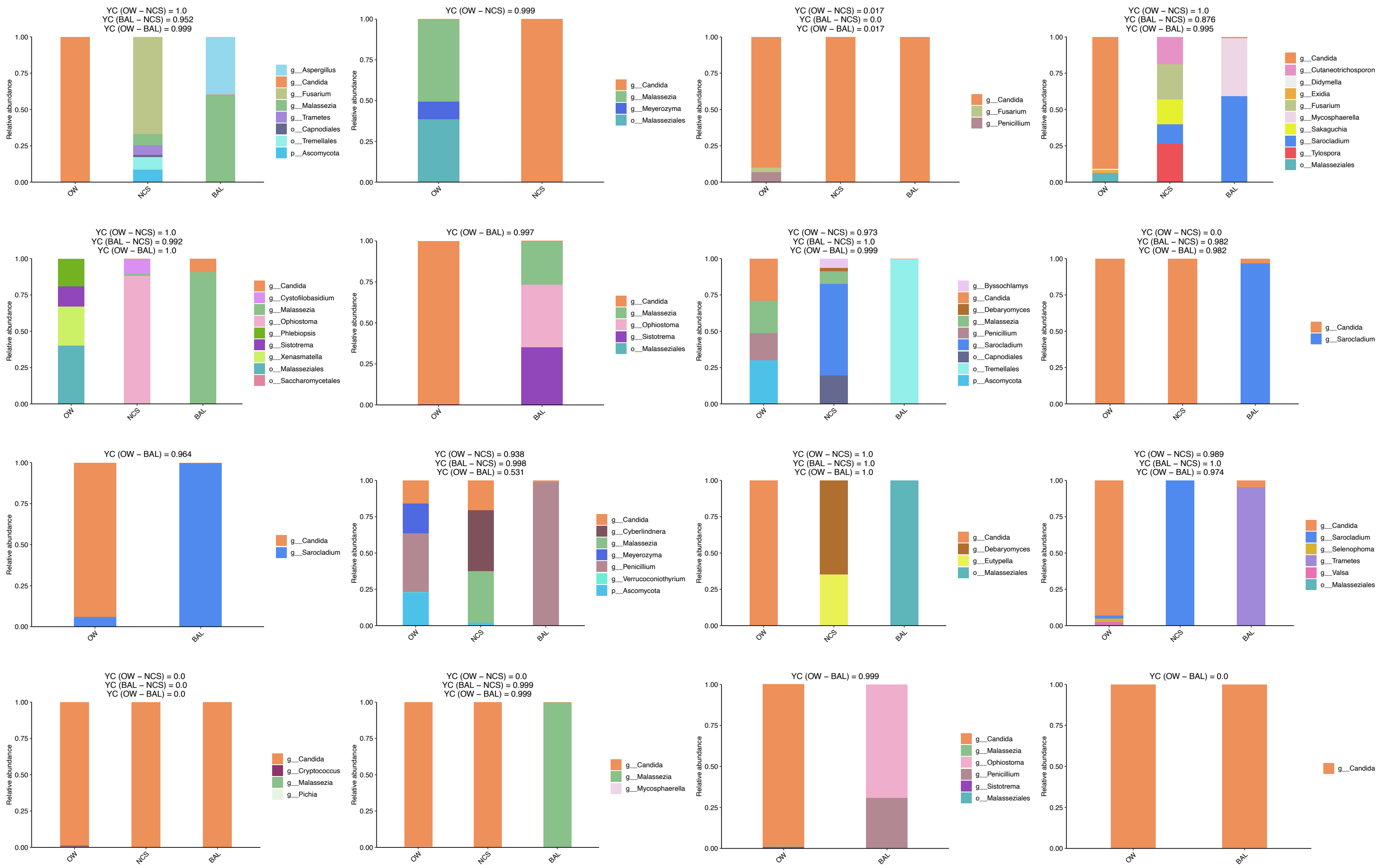
YC (OW – NCS) = 1.0
YC (BAL – NCS) = 0.952
YC (OW – BAL) = 0.999
Relative abundance
OW
NCS
BAL
g__Aspergillus
g__Candida
g__Fusarium
g__Malassezia
g__Trametes
o__Capnodiales
o__Tremellales
p__Ascomycota
YC (OW – NCS) = 0.999
g__Candida
g__Malassezia
g__Meyerozyma
o__Malasseziales
YC (OW – NCS) = 0.017
YC (BAL – NCS) = 0.0
YC (OW – BAL) = 0.017
g__Candida
g__Fusarium
g__Penicillium
YC (OW – NCS) = 1.0
YC (BAL – NCS) = 0.876
YC (OW – BAL) = 0.995
g__Candida
g__Cutaneotrichosporon
g__Didymella
g__Exidia
g__Fusarium
g__Mycosphaerella
g__Sakaguchia
g__Sarocladium
g__Tylospora
o__Malasseziales
YC (OW – NCS) = 1.0
YC (BAL – NCS) = 0.992
YC (OW – BAL) = 1.0
g__Candida
g__Cystofilobasidium
g__Malassezia
g__Ophiostoma
g__Phlebiopsis
g__Sistotrema
g__Xenasmatella
o__Malasseziales
o__Saccharomycetales
YC (OW – BAL) = 0.997
g__Candida
g__Malassezia
g__Ophiostoma
g__Sistotrema
o__Malasseziales
YC (OW – NCS) = 0.973
YC (BAL – NCS) = 1.0
YC (OW – BAL) = 0.999
g__Byssochlamys
g__Candida
g__Debaryomyces
g__Malassezia
g__Penicillium
g__Sarocladium
o__Capnodiales
o__Tremellales
p__Ascomycota
YC (OW – NCS) = 0.0
YC (BAL – NCS) = 0.982
YC (OW – BAL) = 0.982
g__Candida
g__Sarocladium
YC (OW – BAL) = 0.964
g__Candida
g__Sarocladium
YC (OW – NCS) = 0.938
YC (BAL – NCS) = 0.998
YC (OW – BAL) = 0.531
g__Candida
g__Cyberlindnera
g__Malassezia
g__Meyerozyma
g__Penicillium
g__Verrucoconiothyrium
p__Ascomycota
YC (OW – NCS) = 1.0
YC (BAL – NCS) = 1.0
YC (OW – BAL) = 1.0
g__Candida
g__Debaryomyces
g__Eutypella
o__Malasseziales
YC (OW – NCS) = 0.989
YC (BAL – NCS) = 1.0
YC (OW – BAL) = 0.974
g__Candida
g__Sarocladium
g__Selenophoma
g__Trametes
g__Valsa
o__Malasseziales
YC (OW – NCS) = 0.0
YC (BAL – NCS) = 0.0
YC (OW – BAL) = 0.0
g__Candida
g__Cryptococcus
g__Malassezia
g__Pichia
YC (OW – NCS) = 0.0
YC (BAL – NCS) = 0.999
YC (OW – BAL) = 0.999
g__Candida
g__Malassezia
g__Mycosphaerella
YC (OW – BAL) = 0.999
g__Candida
g__Malassezia
g__Ophiostoma
g__Penicillium
g__Sistotrema
o__Malasseziales
YC (OW – BAL) = 0.0
g__Candida

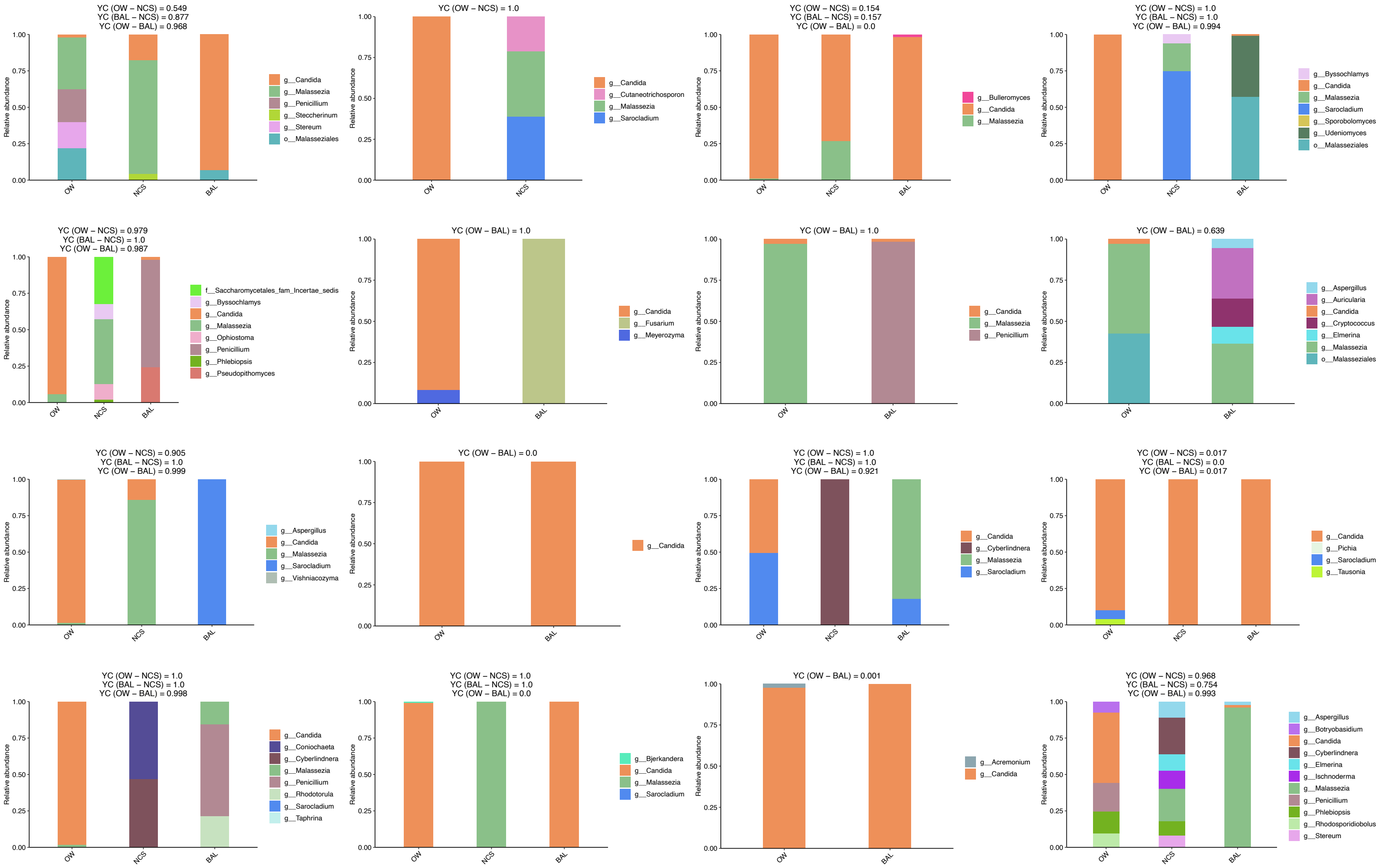
YC (OW – NCS) = 0.549
YC (BAL – NCS) = 0.877
YC (OW – BAL) = 0.968
g__Candida
g__Malassezia
g__Penicillium
g__Steccherinum
g__Stereum
o__Malasseziales
YC (OW – NCS) = 1.0
g__Candida
g__Cutaneotrichosporon
g__Malassezia
g__Sarocladium
YC (OW – NCS) = 0.154
YC (BAL – NCS) = 0.157
YC (OW – BAL) = 0.0
g__Bulleromyces
g__Candida
g__Malassezia
YC (OW – NCS) = 1.0
YC (BAL – NCS) = 1.0
YC (OW – BAL) = 0.994
g__Byssochlamys
g__Candida
g__Malassezia
g__Sarocladium
g__Sporobolomyces
g__Udeniomyces
o__Malasseziales
YC (OW – NCS) = 0.979
YC (BAL – NCS) = 1.0
YC (OW – BAL) = 0.987
f__Saccharomycetales_fam_Incertae_sedis
g__Byssochlamys
g__Candida
g__Malassezia
g__Ophiostoma
g__Penicillium
g__Phlebiopsis
g__Pseudopithomyces
YC (OW – BAL) = 1.0
g__Candida
g__Fusarium
g__Meyerozyma
YC (OW – BAL) = 1.0
g__Candida
g__Malassezia
g__Penicillium
YC (OW – BAL) = 0.639
g__Aspergillus
g__Auricularia
g__Candida
g__Cryptococcus
g__Elmerina
g__Malassezia
o__Malasseziales
YC (OW – NCS) = 0.905
YC (BAL – NCS) = 1.0
YC (OW – BAL) = 0.999
g__Aspergillus
g__Candida
g__Malassezia
g__Sarocladium
g__Vishniacozyma
YC (OW – BAL) = 0.0
g__Candida
YC (OW – NCS) = 1.0
YC (BAL – NCS) = 1.0
YC (OW – BAL) = 0.921
g__Candida
g__Cyberlindnera
g__Malassezia
g__Sarocladium
YC (OW – NCS) = 0.017
YC (BAL – NCS) = 0.0
YC (OW – BAL) = 0.017
g__Candida
g__Pichia
g__Sarocladium
g__Tausonia
YC (OW – NCS) = 1.0
YC (BAL – NCS) = 1.0
YC (OW – BAL) = 0.998
g__Candida
g__Coniochaeta
g__Cyberlindnera
g__Malassezia
g__Penicillium
g__Rhodotorula
g__Sarocladium
g__Taphrina
YC (OW – NCS) = 1.0
YC (BAL – NCS) = 1.0
YC (OW – BAL) = 0.0
g__Bjerkandera
g__Candida
g__Malassezia
g__Sarocladium
YC (OW – BAL) = 0.001
g__Acremonium
g__Candida
YC (OW – NCS) = 0.968
YC (BAL – NCS) = 0.754
YC (OW – BAL) = 0.993
g__Aspergillus
g__Botryobasidium
g__Candida
g__Cyberlindnera
g__Elmerina
g__Ischnoderma
g__Malassezia
g__Penicillium
g__Phlebiopsis
g__Rhodosporidiobolus
g__Stereum
Relative abundance
OW
NCS
BAL

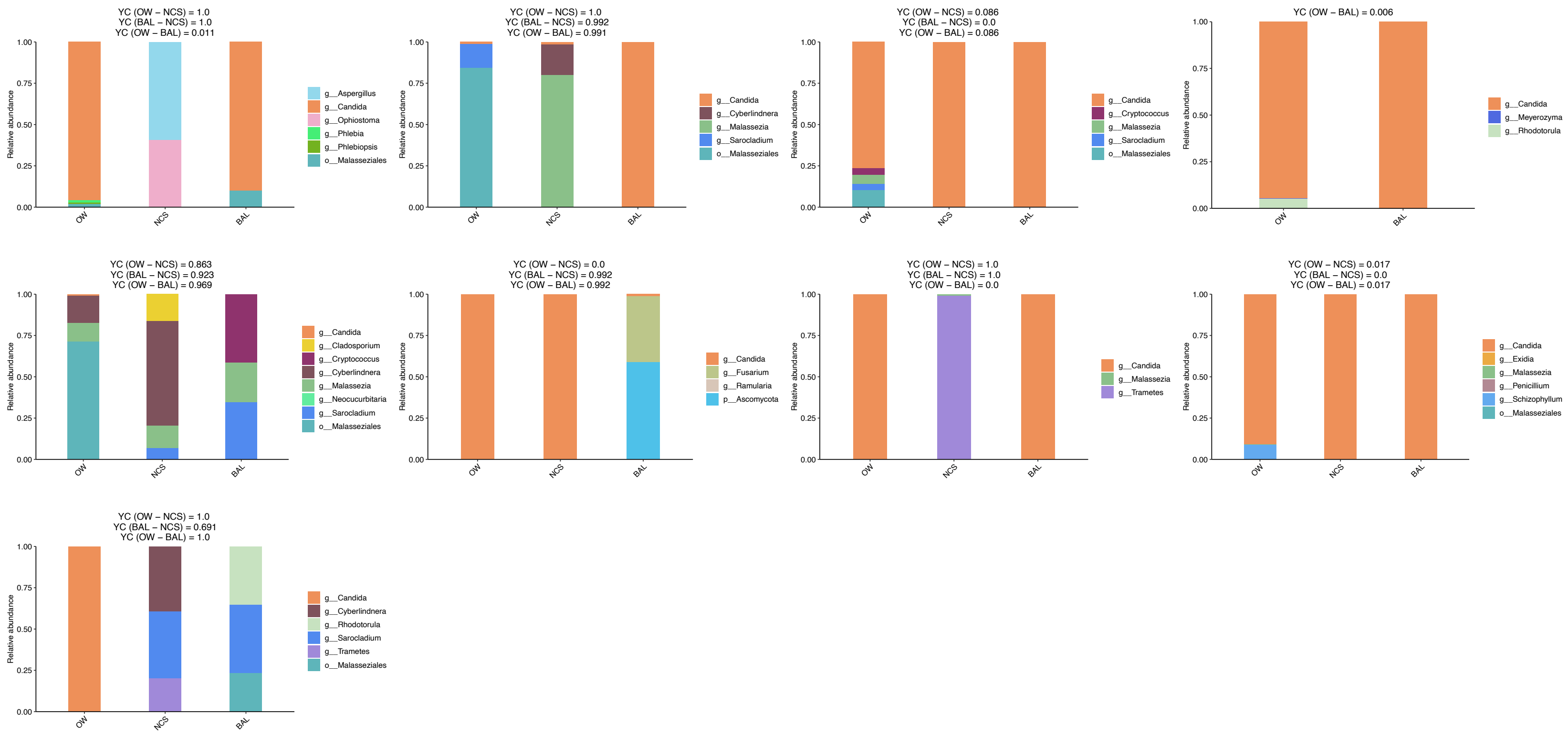

YC: Yue-Clayton measure. OW: oral wash, NCS: negative control sample BAL: bronchoalveolar lavage. A Yue-Clayton measure of 0 means identical sample pairs, while a Yue-Clayton measure of 1 means unidentical sample pairs.
