# Supplementary material for: The pulmonary mycobiome—A study of subjects with and without chronic obstructive pulmonary disease
Source: PLoS One. 2021 Apr 7;16(4):e0248967. doi: 10.1371/journal.pone.0248967 (PMC8026037; doi:10.1371/journal.pone.0248967)
Supplement: S3 Fig — Principal coordinates analysis plots by (A) study group and (B) inhaled steroids use. Differences in beta diversity were tested with permuted analysis of variance (PERMANOVA) adjusted for sex, age, and percentage of predicted FEV1 (permutations = 10000). No significant differences were seen in spread/dispersion (permutations = 1000). (PDF) [file pone.0248967.s003.pdf]

The pulmonary mycobiome - a study of subjects with and without chronic obstructive pulmonary disease

Supporting Information, S3 Fig

Einar M. H. Martinsen[1*], Tomas M. L. Eagan[1,2], Elise O. Leiten[1], Ingvild Haaland[1], Gunnar R. Husebø[1,2], Kristel S. Knudsen[2], Christine Drengenes[1,2], Walter Sanseverino[3], Andreu Paytuví-Gallart[3], and Rune Nielsen[1,2]

[1]Department of Clinical Science, University of Bergen, Bergen, Norway

[2]Department of Thoracic Medicine, Haukeland University Hospital, Bergen, Norway

[3]Sequentia Biotech SL, Barcelona, Spain

* Corresponding author

E-mail: einar.martinsen@uib.no

S3 Fig. Principal coordinates analysis plots by (A) study group and (B) inhaled steroids use.

(A)

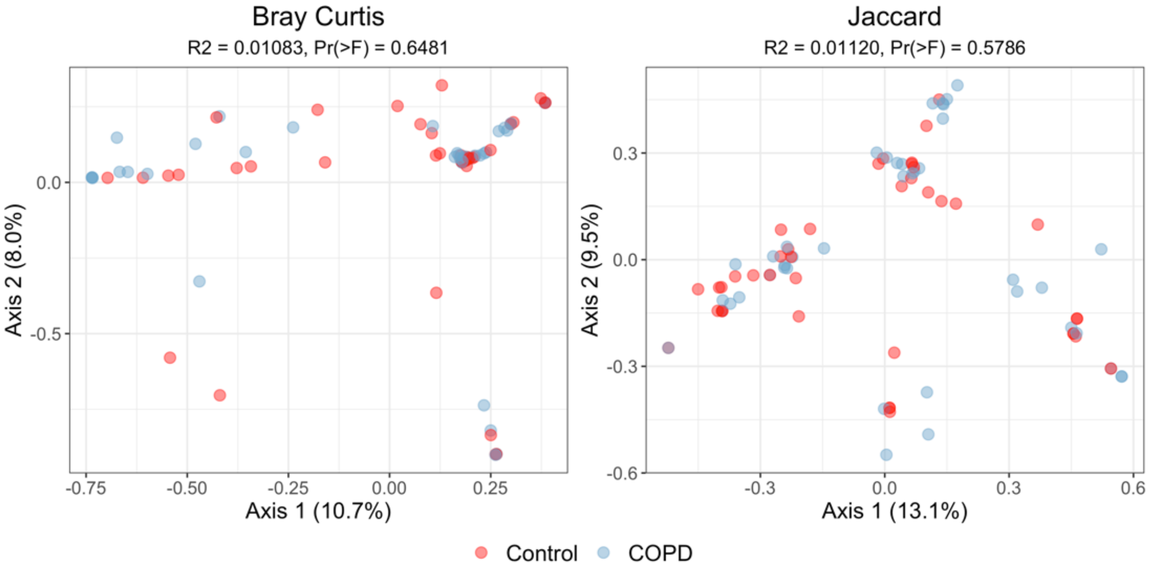

(B)

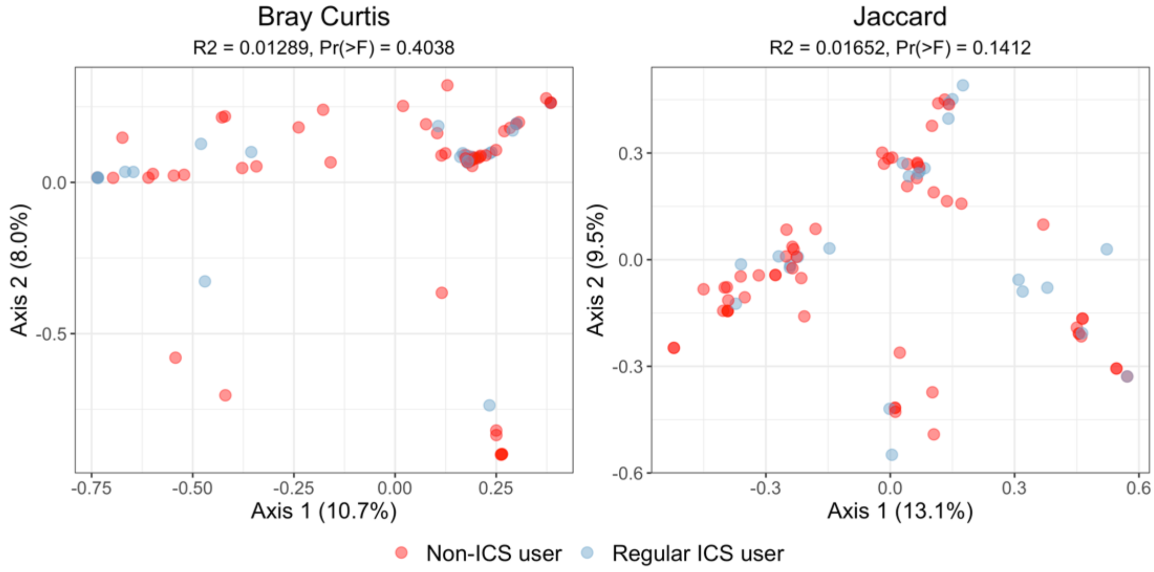

Differences in beta diversity were tested with permuted analysis of variance (PERMANOVA) adjusted for sex, age, and percentage of predicted FEV_1 (permutations = 10000). No significant differences were seen in spread/dispersion (permutations = 1000).
