# Supplementary material for: The pulmonary mycobiome—A study of subjects with and without chronic obstructive pulmonary disease
Source: PLoS One. 2021 Apr 7;16(4):e0248967. doi: 10.1371/journal.pone.0248967 (PMC8026037; doi:10.1371/journal.pone.0248967)
Supplement: S4 Fig — Principal coordinates analysis plots by sample type (A) before and (B) after symmetric Procrustes transformation. OW: oral wash, BAL: bronchoalveolar lavage. Arrows are drawn from the OW sample to the BAL sample from the same participant. Non-randomness (“significance”) between the two configurations was tested with the protest function including the three first axis from the PCoA and specifying 999 permutations. (PDF) [file pone.0248967.s004.pdf]

The pulmonary mycobiome - a study of subjects with and without chronic obstructive pulmonary disease

Supporting Information, S4 Fig

Einar M. H. Martinsen[1*], Tomas M. L. Eagan[1,2], Elise O. Leiten[1], Ingvild Haaland[1], Gunnar R. Husebø[1,2], Kristel S. Knudsen[2], Christine Drengenes[1,2], Walter Sanseverino[3], Andreu Paytuví-Gallart[3], and Rune Nielsen[1,2]

[1]Department of Clinical Science, University of Bergen, Bergen, Norway

[2]Department of Thoracic Medicine, Haukeland University Hospital, Bergen, Norway

[3]Sequentia Biotech SL, Barcelona, Spain

* Corresponding author

E-mail: einar.martinsen@uib.no

S4 Fig. Principal coordinates analysis plots by sample type (A) before and (B) after symmetric Procrustes transformation.

(A)

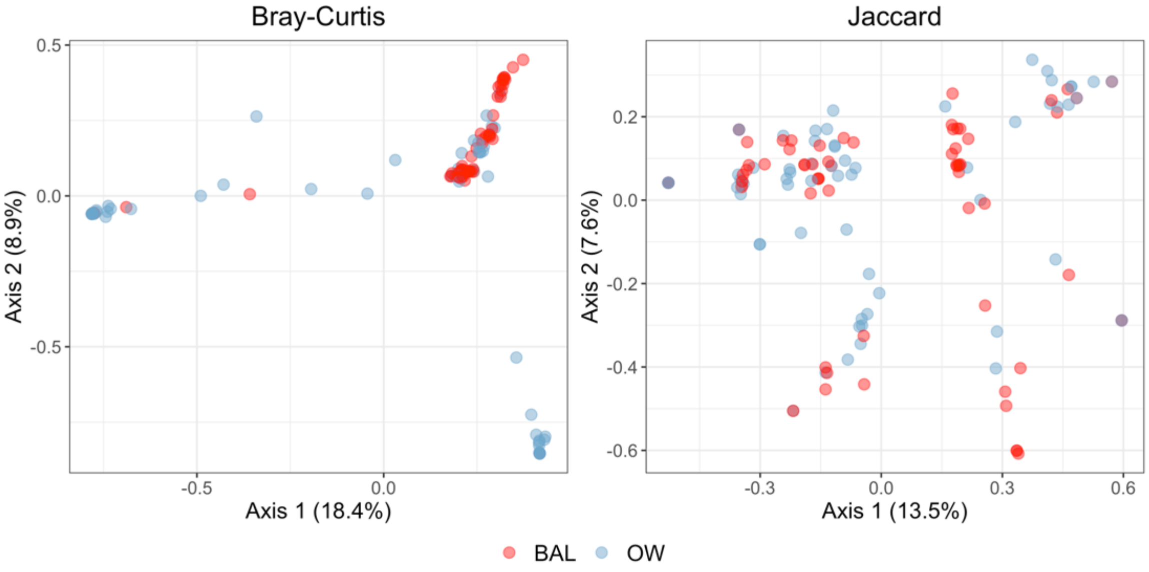

(B)

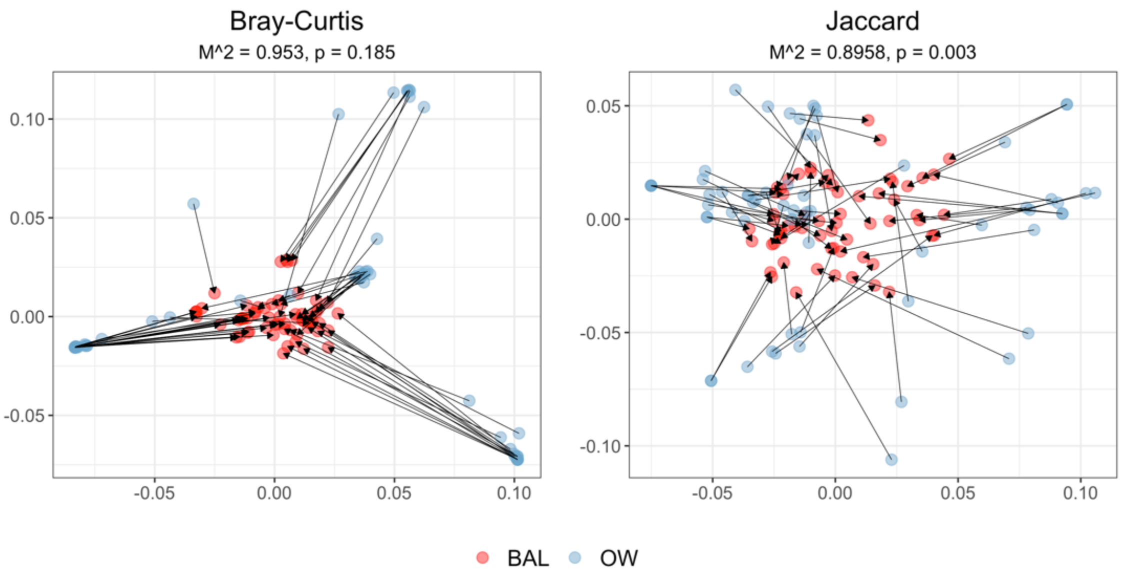

OW: oral wash, BAL: bronchoalveolar lavage. Arrows are drawn from the OW sample to the BAL sample from the same participant. Non-randomness (“significance”) between the two configurations was tested with the protest function including the three first axis from the PCoA and specifying 999 permutations.
