# Supplementary material for: The pulmonary mycobiome—A study of subjects with and without chronic obstructive pulmonary disease
Source: PLoS One. 2021 Apr 7;16(4):e0248967. doi: 10.1371/journal.pone.0248967 (PMC8026037; doi:10.1371/journal.pone.0248967)
Supplement: S5 Fig — BAL: bronchoalveolar lavage, NCS: negative control sample, OW: oral wash. Statistical differences in Qubit concentrations were tested using Wilcoxon signed-rank test as a paired test. (PDF) [file pone.0248967.s005.pdf]

# The pulmonary mycobiome - a study of subjects with and without chronic obstructive pulmonary disease

**Supporting Information, S5 Fig**

Einar M. H. Martinsen[1*], Tomas M. L. Eagan[1,2], Elise O. Leiten[1], Ingvild Haaland[1], Gunnar R. Husebø[1,2], Kristel S. Knudsen[2], Christine Drengenes[1,2], Walter Sanseverino[3], Andreu Paytuví-Gallart[3], and Rune Nielsen[1,2]

[1]Department of Clinical Science, University of Bergen, Bergen, Norway

[2]Department of Thoracic Medicine, Haukeland University Hospital, Bergen, Norway

[3]Sequentia Biotech SL, Barcelona, Spain

* Corresponding author

E-mail: einar.martinsen@uib.no

**S5 Fig. Plot of Qubit concentrations and comparisons between sample types.**

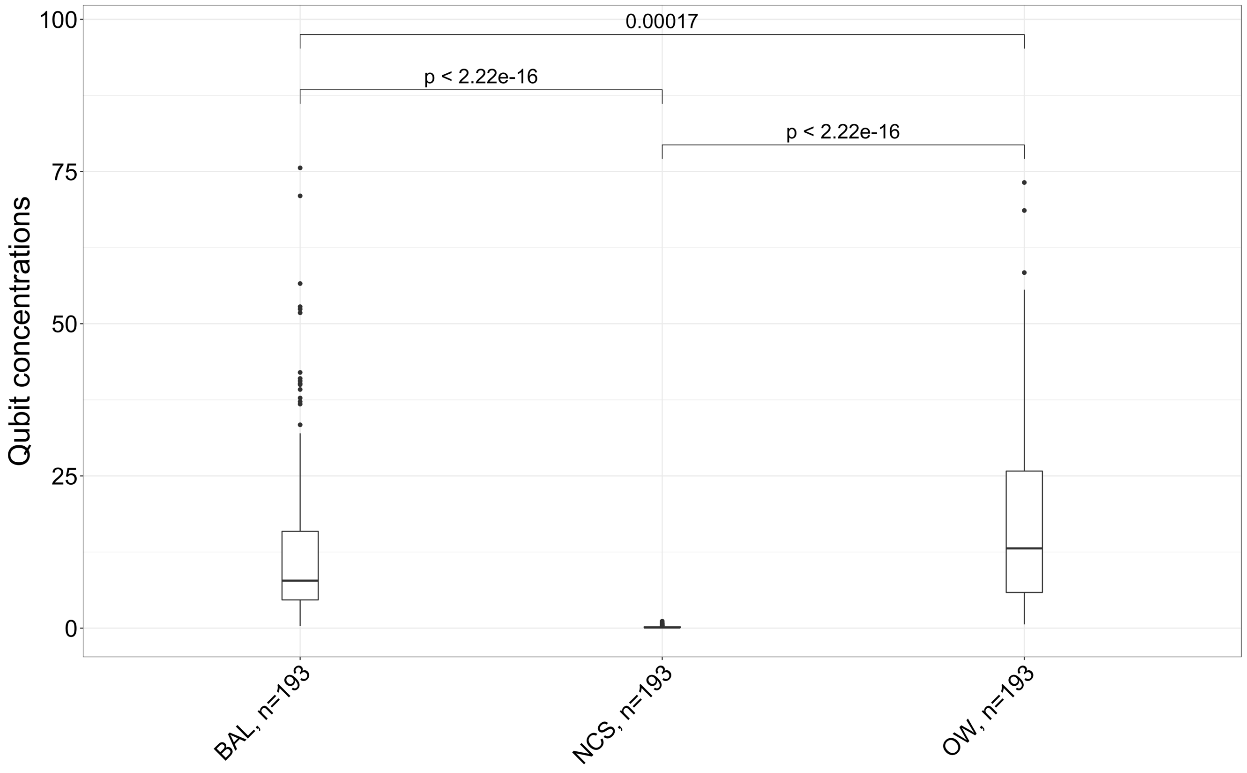


BAL: bronchoalveolar lavage, NCS: negative control sample, OW: oral wash. Statistical differences in Qubit concentrations were tested using Wilcoxon signed-rank test as a paired test.
