# Supplementary material for: The pulmonary mycobiome—A study of subjects with and without chronic obstructive pulmonary disease
Source: PLoS One. 2021 Apr 7;16(4):e0248967. doi: 10.1371/journal.pone.0248967 (PMC8026037; doi:10.1371/journal.pone.0248967)
Supplement: S1 Table — ASV: amplicon sequence variant. The R package “Decontam” identified the ASV IDs above as contaminants. ASVs presumed to be contaminants were removed prior to analyses. (PDF) [file pone.0248967.s008.pdf]

The pulmonary mycobiome - a study of subjects with and without chronic obstructive pulmonary disease

Supporting Information, S1 Table

Einar M. H. Martinsen[1*], Tomas M. L. Eagan[1,2], Elise O. Leiten[1], Ingvild Haaland[1], Gunnar R. Husebø[1,2], Kristel S. Knudsen[2], Christine Drengenes[1,2], Walter Sanseverino[3], Andreu Paytuví-Gallart[3], and Rune Nielsen[1,2]

[1]Department of Clinical Science, University of Bergen, Bergen, Norway

[2]Department of Thoracic Medicine, Haukeland University Hospital, Bergen, Norway

[3]Sequentia Biotech SL, Barcelona, Spain

* Corresponding author

E-mail: einar.martinsen@uib.no

S1 Table. Presumed fungal contaminants identified by Decontam in the MicroCOPD study.

ASV ID	Taxonomic assignment
0530432a79d64c14e54b3194fde23ef0	k__Fungi;p__Ascomycota;c__Dothideomycetes;o__Capnodiales;f__Cladosporiaceae;g__Cladosporium;s__Cladosporium_sphaerospermum
065687e1554cd5393b16eebb205e95db	k__Fungi;p__Basidiomycota;c__Malasseziomycetes;o__Malasseziales;f__Malasseziaceae;g__Malassezia;s__Malassezia_restricta
0977839be912534094022b61487424e3	k__Fungi;p__Basidiomycota;c__Tremellomycetes;o__Cystofilobasidiales;f__Mrakiaceae;g__Itersonilia;s__Itersonilia_perplexans
09a65d7f3915412cf5016bc3ca0ef885	k__Fungi;p__Basidiomycota;c__Malasseziomycetes;o__Malasseziales;f__Malasseziaceae;g__Malassezia;s__Malassezia_arunalokei
16aa8a702afb0c51fad2a4c48c7919a0	k__Fungi;p__Ascomycota;c__Dothideomycetes;o__Pleosporales;f__Sporormiaceae
19ed7271f36279c81e83ffae9238ff76	k__Fungi;p__Basidiomycota;c__Tremellomycetes;o__Trichosporonales;f__Trichosporonaceae;g__Apiotrichum
1ca1c225f9183aee4f5d261493134cb9	k__Fungi;p__Ascomycota;c__Saccharomycetes;o__Saccharomycetales;f__Phaffomycetaceae;g__Cyberlindnera;s__Cyberlindnera_jadinii
2361f6f5271b998d8bf21590f101c232	k__Fungi;p__Ascomycota;c__Saccharomycetes;o__Saccharomycetales;f__Debaryomycetaceae;g__Debaryomyces;s__Debaryomyces_prosopidis
23ed8cd3a2764105e02647ea876d088d	k__Fungi;p__Ascomycota;c__Dothideomycetes;o__Pleosporales;f__Pleosporaceae;g__Curvularia;s__Curvularia_verruculosa
2a3c30645555faae23cc86e0ec21234a	k__Fungi;p__Basidiomycota;c__Malasseziomycetes;o__Malasseziales;f__Malasseziaceae;g__Malassezia;s__Malassezia_restricta
2f171e98ed3a645192767c6adbcf1799	k__Fungi;p__Basidiomycota;c__Agaricomycetes;o__Polyporales;f__Coriolaceae;g__Trametes;s__Trametes_versicolor
309ca8f3329ebf7cd0997896f5a95e4b	k__Fungi;p__Basidiomycota;c__Agaricomycetes;o__Polyporales;f__Steccherinaceae;g__Steccherinum;s__Steccherinum_fimbriatum
31e4540f8df6b5c0acfa08b2f6355586	k__Fungi;p__Ascomycota
338207577405e2abfff92c3279fb65c9	k__Fungi;p__Basidiomycota;c__Agaricomycetes;o__Auriculariales;f__Aporpiaceae;g__Elmerina;s__unidentified
35d98f770540272d21b9f1aeabd3b4e1	k__Fungi;p__Ascomycota
37ab88036d89502720bbc7df1658b874	k__Fungi;p__Ascomycota;c__Dothideomycetes;o__Dothideales;f__Aureobasidiaceae;g__Aureobasidium;s__Aureobasidium_pullulans
3961df9805e9380f4a319e95bf86b1ed	k__Fungi;p__Basidiomycota;c__Malasseziomycetes;o__Malasseziales;f__Malasseziaceae;g__Malassezia;s__Malassezia_restricta
3d5d519c928b4ff559a4d45b52dbb902	k__Fungi;p__Ascomycota;c__Eurotiomycetes;o__Eurotiales;f__Aspergillaceae;g__Aspergillus
3ecaa9fce83dd9b19a6ead5ff061aeaa	k__Fungi;p__Basidiomycota;c__Agaricomycetes;o__Polyporales;f__Coriolaceae;g__Trametes;s__Trametes_cubensis
40deead61013b80520ff52b541559c6a	k__Fungi;p__Ascomycota;c__Saccharomycetes;o__Saccharomycetales;f__Debaryomycetaceae;g__Debaryomyces;s__Debaryomyces_prosopidis
444a3414448a15c1437b92cd857b9063	k__Fungi;p__Basidiomycota;c__Agaricomycetes;o__Polyporales;f__Ischnodermataceae;g__Ischnoderma;s__Ischnoderma_resinosum

46e86fb25f6ec9039c0c9408165d1849	k__Fungi;p__Ascomycota;c__Saccharomycetes;o__Saccharomycetales;f__Saccharomycetales_fam_Incertae_sedis;g__Candida;s__Candida_hyderabadensis
4b80dcdc17d74ad646a811b5edf29b62	k__Fungi;p__Ascomycota;c__Dothideomycetes;o__Capnodiales;f__Dissoconiaceae;g__Uwebraunia;s__Uwebraunia_musae
58e6996017ab0e54ee25470946ac67d5	k__Fungi;p__Ascomycota;c__Sordariomycetes;o__Hypocreales;f__Hypocreaceae;g__Trichoderma;s__Trichoderma_atroviride
5bbad0df8fb4b39cd1605b1ac5c750d2	k__Fungi;p__Ascomycota;c__Saccharomycetes;o__Saccharomycetales;f__Saccharomycetales_fam_Incertae_sedis;g__Candida;s__Candida_orthopsilosis
5c3105c7dae5c3a71cdb52d3d1556c4b	k__Fungi;p__Ascomycota;c__Saccharomycetes;o__Saccharomycetales;f__Saccharomycetales_fam_Incertae_sedis;g__Candida;s__Candida_tropicalis
603623f01f16ccba7e3f3247531d602d	k__Fungi;p__Ascomycota;c__Dothideomycetes;o__Capnodiales
607d9b7d133be405482d3d80ec86caf0	k__Fungi;p__Basidiomycota;c__Malasseziomycetes;o__Malasseziales;f__Malasseziaceae;g__Malassezia;s__Malassezia_arunalokei
670e919f3c18339ed5317dc98f136d1b	k__Fungi;p__Ascomycota
698e5f3db97e4aa8848ff7b0be33b25b	k__Fungi;p__Ascomycota;c__Eurotiomycetes;o__Eurotiales;f__Aspergillaceae;g__Aspergillus;s__Aspergillus_conicus
6b6fd21593f67cac437da89ceedd42af	k__Fungi;p__Ascomycota;c__Eurotiomycetes;o__Eurotiales;f__Thermoascaceae;g__Byssochlamys;s__Byssochlamys_laguncularia e
6c558ecd0cec0caa6047e3530f9d84dd	k__Fungi;p__Ascomycota;c__Eurotiomycetes;o__Eurotiales;f__Aspergillaceae;g__Aspergillus;s__Aspergillus_penicillioides
6f723fff4a53380954cc5d5705b4bbfe	k__Fungi;p__Basidiomycota;c__Agaricomycetes;o__Polyporales;f__Meruliaceae;g__Phlebia;s__Phlebia_tremellosa
7097950963a5a0a3ff900475cc8daf06	k__Fungi;p__Basidiomycota;c__Tremellomycetes;o__Filobasidiales;f__Filobasidiaceae;g__Naganishia;s__Naganishia_globosa
71bf558aba72ec5cbd9a6446659959c8	k__Fungi;p__Basidiomycota;c__Cystobasidiomycetes;o__Erythrobasidiales;f__Erythrobasidiales_fam_Incertae_sedis;g__Sakaguchia;s__unidentified
722db407e71223e0568d98d333749c4d	k__Fungi;p__Ascomycota;c__Eurotiomycetes;o__Eurotiales;f__Aspergillaceae;g__Aspergillus;s__Aspergillus_ruber
771ff9789acfcf29b25c753fd743418c	k__Fungi;p__Ascomycota;c__Eurotiomycetes;o__Eurotiales;f__Thermoascaceae;g__Byssochlamys;s__Byssochlamys_lagunculariae
783b7b48c8ff0ad7572121325efac642	k__Fungi;p__Basidiomycota;c__Agaricomycetes;o__Polyporales;f__Meruliaceae;g__Irpex;s__Irpex_hydnoides
7a6657d7cf9fb7066260b2fe1653912b	k__Fungi;p__Basidiomycota;c__Malasseziomycetes;o__Malasseziales;f__Malasseziaceae;g__Malassezia;s__Malassezia_globosa
7af3d5ac035c84b7d7a573684ae981f6	k__Fungi;p__Ascomycota;c__Eurotiomycetes;o__Eurotiales;f__Aspergillaceae;g__Aspergillus;s__Aspergillus_subversicolor
86fed379f41d9e5ba162c5124ac0c116	k__Fungi;p__Ascomycota;c__Saccharomycetes;o__Saccharomycetales;f__Saccharomycetales_fam_Incertae_sedis;g__Candida;s__Candida_albicans
8a2ac77b455f32571129350a1af4a0a2	k__Fungi;p__Ascomycota;c__Eurotiomycetes;o__Chaetothyriales;f__Trichomeriaceae;g__unidentified;s__unidentified
8c105f1ef02b3a9e62202d1ac0f45af9	k__Fungi;p__Ascomycota;c__Saccharomycetes;o__Saccharomycetales;f__Saccharomycetales_fam_Incertae_sedis;g__Candida;s__Candida_dubliniensis
8cee11e801c97830c0021ca9b520ac35	k__Fungi;p__Ascomycota;c__Eurotiomycetes;o__Eurotiales;f__Aspergillaceae;g__Penicillium;s__Penicillium_coprobium

8f9ec8d60885bca69f4582eddfb6b6cf	k__Fungi;p__Basidiomycota;c__Agaricomycetes;o__Cantharellales;f__Cantharellales_fam_Incertae_sedis;g__Sistotrema;s__Sistotrema_sernanderi
91e1a5937dd11841ffa372e09d7b9499	k__Fungi;p__Basidiomycota;c__Tremellomycetes;o__Trichosporonales;f__Trichosporonaceae;g__Cutaneotrichosporon
9381379d0ba83b70bf195275193c89e7	k__Fungi;p__Ascomycota;c__Eurotiomycetes;o__Chaetothyriales;f__Herpotrichiellaceae;g__Coniosporium;s__Coniosporium_apollinis
996905c1f73ab9d202da2f2f601d74aa	k__Fungi;p__Basidiomycota;c__Malasseziomycetes;o__Malasseziales;f__Malasseziaceae;g__Malassezia
a0cd5c00136da7059ca5de6e79d97a58	k__Fungi;p__Basidiomycota;c__Malasseziomycetes;o__Malasseziales;f__Malasseziaceae;g__Malassezia
a2a7ba33c4004db4f00b3ecfdc735593	k__Fungi;p__Basidiomycota;c__Agaricomycetes;o__Hymenochaetales;f__Hymenochaetaceae;g__Phellinus;s__Phellinus_gilvus
a33767266c69be3d2c2646d1ca52b152	k__Fungi;p__Basidiomycota;c__Microbotryomycetes;o__Sporidiobolales;f__Sporidiobolaceae;g__Rhodosporidiobolus;s__Rhodosporidiobolus_colostri
a3975503fd1fe1c5843575e245af495c	k__Fungi;p__Ascomycota;c__Saccharomycetes;o__Saccharomycetales;f__Phaffomycetaceae;g__Cyberlindnera;s__Cyberlindnera_jadinii
a52c220f70e3743a76246d8ac83611d3	k__Fungi;p__Basidiomycota;c__Wallemiomycetes;o__Wallemiales;f__Wallemiaceae;g__Wallemia
a7f00ae8dab4a2afd9c4ceeb2f3816db	k__Fungi;p__Ascomycota
a9507ddcd26034574b4403a79f4a71c6	k__Fungi;p__Basidiomycota;c__Agaricomycetes;o__Polyporales;f__Meruliaceae;g__Ceriporia;s__Ceriporia_alachuana
a9ef62bfa868aade17a5e39b229a6d14	k__Fungi;p__Ascomycota;c__Saccharomycetes;o__Saccharomycetales;f__Saccharomycetales_fam_Incertae_sedis
aa562736d18d9f7abad5ff4aadb089a6	k__Fungi;p__Basidiomycota;c__Microbotryomycetes;o__Sporidiobolales;f__Sporidiobolaceae;g__Rhodosporidiobolus;s__unidentified
af04f0bfa7a370b8ebddcd050562f5fd	k__Fungi;p__Ascomycota;c__Saccharomycetes;o__Saccharomycetales;f__Saccharomycetales_fam_Incertae_sedis;g__Candida;s__Candida_albicans
b42d6fe1e4f5cb7ed02698396b87968e	k__Fungi;p__Ascomycota;c__Dothideomycetes;o__Pleosporales;f__Didymellaceae
b887553771c918a81de1cfeaacdd5c00	k__Fungi;p__Mucoromycota;c__Mucoromycetes;o__Mucorales;f__Rhizopodaceae;g__Rhizopus;s__Rhizopus_arrhizus
c0d02e8cb387d663776e12b19af01038	k__Fungi;p__Basidiomycota;c__Tremellomycetes;o__Filobasidiales;f__Filobasidiaceae;g__Naganishia;s__Naganishia_diffluens
c3f1d634ef920f1e4156afec72978cf5	k__Fungi;p__Ascomycota;c__Dothideomycetes;o__Capnodiales;f__Cladosporiaceae;g__Cladosporium;s__unidentified
c65a5b2edf7a035a23e887d37c143c39	k__Fungi;p__Basidiomycota;c__Tremellomycetes;o__Filobasidiales;f__Filobasidiaceae;g__Filobasidium;s__Filobasidium_magnum
c73189c0e862ddad73885c35fc7f960d	k__Fungi;p__Ascomycota;c__Sordariomycetes;o__Hypocreales;f__Nectriaceae;g__Fusarium
c8badf543e584cbd4e814f8a504db281	k__Fungi;p__Basidiomycota;c__Malasseziomycetes;o__Malasseziales;f__Malasseziaceae;g__Malassezia
cc98c1eaacd168910696b59c7d5b3bc0	k__Fungi;p__Basidiomycota;c__Cystobasidiomycetes;o__Erythrobasidiales;f__Erythrobasidiales_fam_Incertae_sedis;g__Sakaguchia
d10278d5ea5a8a5d15c7690bf0358c19	k__Fungi;p__Ascomycota;c__Sordariomycetes;o__Hypocreales;f__Stachybotryaceae;g__Stachybotrys;s__Stachybotrys_chartarum

d1922a8e9eade2a625643dc88341fd3c	k__Fungi;p__Basidiomycota;c__Wallemiomycetes;o__Wallemiales;f__Wallemiaceae;g__Wallemia
d71c16ae9cb27049bd078cd70477a834	k__Fungi;p__Ascomycota;c__Dothideomycetes;o__Capnodiales
dc19bc19ead2778c4530a5649096bfa3	k__Fungi;p__Ascomycota;c__Saccharomycetes;o__Saccharomycetales;f__Saccharomycetales_fam_Incertae_sedis;g__Candida;s__Candida_tropicalis
e0af56eb434e3a071a73172357e2d47b	k__Fungi;p__Ascomycota;c__Dothideomycetes;o__Pleosporales;f__Didymellaceae
e1d5c562010b135d34d985fd1ce89c2b	k__Fungi;p__Ascomycota;c__Sordariomycetes;o__Diaporthales;f__Diaporthaceae;g__Diaporthe;s__Diaporthe_kochmanii
e280e3a68e44cb2c79ef7eb11a44c00f	k__Fungi
e55180172dd14de49ee78e76629b7eff	k__Fungi;p__Ascomycota;c__Eurotiomycetes;o__Eurotiales;f__Aspergillaceae;g__Penicillium
e79a39e2aae24009f58fb2d3c971a386	k__Fungi;p__Ascomycota;c__Saccharomycetes;o__Saccharomycetales;f__Phaffomycetaceae;g__Cyberlindnera;s__Cyberlindnera_jadinii
e8989580e254cd34bed19fe5ae32289f	k__Fungi;p__Ascomycota;c__Dothideomycetes;o__Capnodiales
e9d30e16cace550de466f0b9ae79ed1b	k__Fungi;p__Ascomycota;c__Dothideomycetes;o__Pleosporales;f__Pleosporaceae;g__Alternaria;s__unidentified
e9eade82a580df375bdc952b77f40527	k__Fungi;p__Basidiomycota;c__Agaricomycetes;o__Russulales;f__Stereaceae;g__Stereum;s__Stereum_complicatum
ed5307ccaa2df99d6b613b468c58dbcb	k__Fungi;p__Basidiomycota;c__Malasseziomycetes;o__Malasseziales;f__Malasseziaceae;g__Malassezia;s__Malassezia_restricta

ASV: amplicon sequence variant. The R package "Decontam" identified the ASV IDs above as contaminants. ASVs presumed to be contaminants were removed prior to analyses.
