# Supplementary material for: The pulmonary mycobiome—A study of subjects with and without chronic obstructive pulmonary disease
Source: PLoS One. 2021 Apr 7;16(4):e0248967. doi: 10.1371/journal.pone.0248967 (PMC8026037; doi:10.1371/journal.pone.0248967)
Supplement: S2 Table — ANCOM v2: the second version of analysis of composition of microbiomes, MicrobiomeDDA: Microbiome Differential Distribution Analysis omnibus test, ALDEx2: the second version of ANOVA-Like Differential Expression, OW: oral wash, BAL: bronchoalveolar lavage. The most conservative value in ANCOM v2 has been used in the analyses (i.e. 0.9). Significance level = 0.05. Never- and ex-smokers were merged into non-smokers. The ALDEx2 approach works poorly if there are only a small number of taxa (less than about 50), so some groups were not analysed. (PDF) [file pone.0248967.s009.pdf]

The pulmonary mycobiome - a study of subjects with and without chronic obstructive pulmonary disease

Supporting Information, S2 Table

Einar M. H. Martinsen[1*], Tomas M. L. Eagan[1,2], Elise O. Leiten[1], Ingvild Haaland[1], Gunnar R. Husebø[1,2], Kristel S. Knudsen[2], Christine Drengenes[1,2], Walter Sanseverino[3], Andreu Paytuví-Gallart[3], and Rune Nielsen[1,2]

[1]Department of Clinical Science, University of Bergen, Bergen, Norway

[2]Department of Thoracic Medicine, Haukeland University Hospital, Bergen, Norway

[3]Sequentia Biotech SL, Barcelona, Spain

* Corresponding author

E-mail: einar.martinsen@uib.no

S2 Table. Differential abundance/distribution testing on fungi in the MicroCOPD study using ANCOM v2, MicrobiomeDDA, and ALDEx2.

Data	ANCOM v2	MicrobiomeDDA	ALDEx2
**Control**			
OW vs BAL	Candida	Not applicable on paired data	Candida
OW vs BAL, smokers	Candida	Not applicable on paired data	Too few taxa
OW vs BAL, non-smokers	Candida	Not applicable on paired data	Candida
**COPD**			
OW vs BAL	Candida	Not applicable on paired data	Candida
OW vs BAL, smokers	Candida	Not applicable on paired data	Too few taxa
OW vs BAL, non-smokers	Candida	Not applicable on paired data	Candida
OW vs BAL, ICS-users	Candida	Not applicable on paired data	Candida
OW vs BAL, non-ICS users	Candida	Not applicable on paired data	Too few taxa
**All study groups**			
Control vs COPD, OW	No significant taxa	Malasseziales, Meyerozyma	No significant taxa
Control vs COPD, BAL	No significant taxa	Penicillium	No significant taxa

ANCOM v2: the second version of analysis of composition of microbiomes, MicrobiomeDDA: Microbiome Differential Distribution Analysis omnibus test, ALDEx2: the second version of ANOVA-Like Differential Expression, OW: oral wash, BAL: bronchoalveolar lavage. The most conservative value in ANCOM v2 has been used in the analyses (i.e. 0.9). Significance level=0.05. Never- and ex-smokers were merged into non-smokers. The ALDEx2 approach works poorly if there are only a small number of taxa (less than about 50), so some groups were not analysed.
