# Supplementary material for: The pulmonary mycobiome—A study of subjects with and without chronic obstructive pulmonary disease
Source: PLoS One. 2021 Apr 7;16(4):e0248967. doi: 10.1371/journal.pone.0248967 (PMC8026037; doi:10.1371/journal.pone.0248967)
Supplement: S3 Table — PERMANOVA: permuted analysis of variance, OW: oral wash, BAL: bronchoalveolar lavage, AN: ANCOM v2, M: MicrobiomeDDA, AL: ALDEx2, sign: significant, FEV1: forced expiratory volume in 1 second. Analyses on FEV1 were omitted for each study group separately due to a majority of controls having above 80% of predicted, and a majority of participants with COPD having below 80% of predicted. Diversity analyses on smoking habits in BAL samples from controls were omitted due to a lack of current smokers. Analyses on smoking habits were done by comparing current vs non-current smokers. (PDF) [file pone.0248967.s010.pdf]

# The pulmonary mycobiome - a study of subjects with and without chronic obstructive pulmonary disease

**Supporting Information, S3 Table**

Einar M. H. Martinsen[1*], Tomas M. L. Eagan[1,2], Elise O. Leiten[1], Ingvild Haaland[1], Gunnar R. Husebø[1,2], Kristel S. Knudsen[2], Christine Drengenes[1,2], Walter Sanseverino[3], Andreu Paytuví-Gallart[3], and Rune Nielsen[1,2]

[1]Department of Clinical Science, University of Bergen, Bergen, Norway

[2]Department of Thoracic Medicine, Haukeland University Hospital, Bergen, Norway

[3]Sequentia Biotech SL, Barcelona, Spain

* Corresponding author

E-mail: einar.martinsen@uib.no

## S3 Table. Taxonomy and diversity comparisons of selected clinical variables in the MicroCOPD study divided by sample type and study group.

| Data | Differential abundance/distribution testing | | Alpha diversity (Shannon index) | | Beta diversity (PERMANOVA) | |
|---|---|---|---|---|---|---|
| | **OW** | **BAL** | **OW** | **BAL** | **OW** | **BAL** |
| **Control** | | | | | | |
| Sex | AN, M, AL: No sign taxa | AN, AL: No sign taxa<br>M: Ascomycota, Ophiostoma | p-value = 0.8675 | p-value = 0.8597 | BC. R2: 0.0213 p-value: 0.152<br>J. R2: 0.02795 p-value: *0.0136* | BC. R2: 0.01937 p-value: 0.8051<br>J. R2: 0.02422 p-value: 0.5033 |
| Age ( < or ≥ 70 years) | AN, M, AL: No sign taxa | AN, AL: No sign taxa<br>M: Ophiostoma, Penicillium | p-value = 0.5152 | p-value = 0.4792 | BC. R2: 0.0054 p-value: 0.9112<br>J. R2: 0.0083 p-value: 0.9123 | BC. R2: 0.02306 p-value: 0.5942<br>J. R2: 0.01877 p-value: 0.8398 |
| Smoking | AN, AL: No sign taxa<br>M: Ascomycota, Valsa | AN, AL: No sign taxa<br>M: Ophiostoma | p-value = 0.6011 | | BC. Sign dispersion, no PERMANOVA<br>J. R2: 0.00798 p-value: 0.9274 | |
| **COPD** | | | | | | |
| Sex | AN, AL: No sign taxa<br>M: Meyerozyma | AN, AL: No sign/few taxa<br>M: Aspergillus | p-value = 0.6581 | p-value = 0.4095 | BC. R2: 0.00475 p-value: 0.9201<br>J. R2: 0.01088 p-value: 0.6937 | BC. R2: 0.0178 p-value: 0.8536<br>J. R2: 0.01371 p-value: 0.9781 |
| Age ( < or ≥ 70 years) | AN, M, AL: No sign taxa | AN, M, AL: No sign/few taxa | p-value = 0.1117 | p-value = 0.3093 | BC. R2: 0.01114 p-value: 0.4854<br>J. R2: 0.01186 p-value: 0.5912 | BC. R2: 0.01949 p-value: 0.7634<br>J. R2: 0.0245 p-value: 0.4528 |
| Smoking | AN, M, AL: No sign taxa | AN, M, AL: No sign/few taxa | p-value = 0.9348 | p-value = 0.7306 | BC. R2: 0.00707 p-value: 0.7582<br>J. R2: 0.00836 p-value: 0.916 | BC. R2: 0.02417 p-value: 0.4743<br>J. R2: 0.02201 p-value: 0.5854 |
| Inhaled steroids use | AN, M, AL: No sign taxa | AN, M, AL: No sign/few taxa | p-value = 0.6358 | p-value = 0.6619 | BC. R2: 0.00694 p-value: 0.7721<br>J. R2: 0.00536 p-value: 0.9989 | BC. R2: 0.01834 p-value: 0.8196<br>J. R2: 0.02293 p-value: 0.5336 |
| **All study groups** | | | | | | |
| Sex | AN, AL: No sign taxa<br>M: Meyerozyma | AN, AL: No sign taxa<br>M: Ophiostoma | p-value = 0.6178 | p-value = 0.4886 | BC. R2: 0.00813 p-value: 0.2616<br>J. R2: 0.01291 p-value: *0.0263* | BC. R2: 0.00794 p-value: 0.9385<br>J. R2: 0.00859 p-value: 0.8881 |
| Age ( < or ≥ 70 years) | AN, M, AL: No sign taxa | AN, AL: No sign taxa<br>M: Penicillium | p-value = 0.3855 | p-value = 0.1964 | BC. R2: 0.00422 p-value: 0.6483<br>J. R2: 0.00562 p-value: 0.6264 | BC. R2: 0.01022 p-value: 0.7101<br>J. R2: 0.01208 p-value: 0.4694 |
| $FEV_1$, % pred<br>( < or ≥ 80) | AN, AL: No sign taxa<br>M: Malasseziales,<br>Ophiostoma, Meyerozyma | AN, M, AL: No sign taxa | p-value = 0.3192 | p-value = 0.7264 | BC. R2: 0.00804 p-value: 0.2729<br>J. R2: 0.0095 p-value: 0.119 | BC. R2: 0.01177 p-value: 0.5151<br>J. R2: 0.01439 p-value: 0.2468 |
| Smoking | AN, M, AL: No sign taxa | AN, AL: No sign taxa<br>M: Sarocladium | p-value = 0.7606 | p-value = 0.3245 | BC. R2: 0.00897 p-value: 0.2206<br>J. R2: 0.00391 p-value: 0.9409 | BC. R2: 0.01148 p-value: 0.5521<br>J. R2: 0.01099 p-value: 0.5982 |
| Inhaled steroids use | AN, M, AL: No sign taxa | AN, M, AL: No sign taxa | p-value = 0.8595 | p-value = 0.7839 | BC. R2: 0.00249 p-value: 0.9201<br>J. R2: 0.00361 p-value: 0.9673 | BC. R2: 0.01289 p-value: 0.3884<br>J. R2: 0.01652 p-value: 0.1293 |

PERMANOVA: permuted analysis of variance, OW: oral wash, BAL: bronchoalveolar lavage, AN: ANCOM v2, M: MicrobiomeDDA, AL: ALDEx2, sign: significant, $FEV_1$: forced expiratory volume in 1 second. Analyses on $FEV_1$ were omitted for each study group separately due to a majority of controls having above 80% of predicted, and a majority of participants with COPD having below 80% of predicted. Diversity analyses on smoking habits in BAL samples from controls were omitted due to a lack of current smokers. Analyses on smoking habits were done by comparing current vs non-current smokers.
