# Supplementary material for: The pulmonary mycobiome—A study of subjects with and without chronic obstructive pulmonary disease
Source: PLoS One. 2021 Apr 7;16(4):e0248967. doi: 10.1371/journal.pone.0248967 (PMC8026037; doi:10.1371/journal.pone.0248967)
Supplement: S4 Table — NCS: Negative control sample, OW: oral wash, BAL: bronchoalveolar lavage, DADA2: Divisive Amplicon Denoising Algorithm version 2. (PDF) [file pone.0248967.s011.pdf]

The pulmonary mycobiome - a study of subjects with and without chronic obstructive pulmonary disease

Supporting Information, S4 Table

Einar M. H. Martinsen[1*], Tomas M. L. Eagan[1,2], Elise O. Leiten[1], Ingvild Haaland[1], Gunnar R. Husebø[1,2], Kristel S. Knudsen[2], Christine Drengenes[1,2], Walter Sanseverino[3], Andreu Paytuví-Gallart[3], and Rune Nielsen[1,2]

[1]Department of Clinical Science, University of Bergen, Bergen, Norway

[2]Department of Thoracic Medicine, Haukeland University Hospital, Bergen, Norway

[3]Sequentia Biotech SL, Barcelona, Spain

* Corresponding author

E-mail: einar.martinsen@uib.no

S4 Table. Summary of read/sequence counts in the MicroCOPD study.

Data	Number of samples	Read/sequence count	Mean read/sequence count
**Raw data**			
NCS	189	667,033	3,529
OW	193	22,933,493	118,826
BAL	193	20,348,685	105,434
**Post ITSxpress**			
NCS	184	529,519	2,878
OW	193	11,907,308	61,696
BAL	193	5,790,865	30,004
**Post DADA2**			
NCS	161	472,574	2,935
OW	193	11,236,458	58,220
BAL	193	5,317,673	27,553
**Post LULU**			
NCS	157	410,205	2,613
OW	193	10,881,332	56,380
BAL	192	4,300,589	22,399
**Without low abundance and contaminants**			
NCS	122	73,658	604
OW	192	9,490,666	49,431
BAL	185	1,663,488	8,992

NCS: Negative control sample, OW: oral wash, BAL: bronchoalveolar lavage, DADA2: Divisive Amplicon Denoising Algorithm version 2.
