# Supplementary material for: The pulmonary mycobiome—A study of subjects with and without chronic obstructive pulmonary disease
Source: PLoS One. 2021 Apr 7;16(4):e0248967. doi: 10.1371/journal.pone.0248967 (PMC8026037; doi:10.1371/journal.pone.0248967)
Supplement: S5 Table — Comparisons were done (A) merged and (B) pairwise. OW: oral wash, BAL: bronchoalveolar lavage, yrs: years. Differences in beta diversity were tested with permuted analysis of variance (PERMANOVA) adjusted for sample type, study group, sex, and age (permutations = 10000). (PDF) [file pone.0248967.s012.pdf]

The pulmonary mycobiome - a study of subjects with and without chronic obstructive pulmonary disease

Supporting Information, S5 Table

Einar M. H. Martinsen[1*], Tomas M. L. Eagan[1,2], Elise O. Leiten[1], Ingvild Haaland[1], Gunnar R. Husebø[1,2], Kristel S. Knudsen[2], Christine Drengenes[1,2], Walter Sanseverino[3], Andreu Paytuví-Gallart[3], and Rune Nielsen[1,2]

[1]Department of Clinical Science, University of Bergen, Bergen, Norway

[2]Department of Thoracic Medicine, Haukeland University Hospital, Bergen, Norway

[3]Sequentia Biotech SL, Barcelona, Spain

* Corresponding author

E-mail: einar.martinsen@uib.no

S5 Table. Beta diversity comparisons using Bray-Curtis and Jaccard distances. Comparisons were done (A) merged and (B) pairwise.

(A)

Bray-Curtis, adjusted PERMANOVA on OW and BAL together						
**Variables**	**Df**	**Sum of squares**	**Mean squares**	**F.Model**	**R2**	**Pr(>F)**
Sequencing run	2	2.324	1.1618	3.0858	*0.02459*	*0.0001*
Sample type	1	7.733	7.7328	20.5389	*0.08183*	*0.0001*
Study group	1	0.401	0.4008	1.0646	0.00424	0.3369
Sex	1	0.437	0.4374	1.1618	0.00463	0.2667
Age (10 yrs)	5	1.153	0.2305	0.6123	0.0122	0.9898
Residuals	219	82.452	0.3765	-	0.87252	-
Total	229	94.499	-	-	1	-
**Jaccard, adjusted PERMANOVA on OW and BAL together**						
**Variables**	**Df**	**Sum of squares**	**Mean squares**	**F.Model**	**R2**	**Pr(>F)**
Sequencing run	2	4.34	2.1699	5.8357	*0.04834*	*0.0001*
Sample type	1	1.309	1.30855	3.5192	*0.01457*	*0.0002*
Study group	1	0.536	0.53639	1.4426	0.00597	0.0987
Sex	1	0.498	0.49847	1.3406	0.00555	0.1306
Age (10 yrs)	5	1.669	0.33379	0.8977	0.01859	0.7540
Residuals	219	81.431	0.37183	-	0.90697	-
Total	229	89.784	-	-	1	-

(B)

Bray-Curtis, pairwise, adjusted PERMANOVA on OW and BAL together<br>Sequencing run 1 and 2						
**Variables**	**Df**	**Sum of squares**	**Mean squares**	**F.Model**	**R2**	**Pr(>F)**
Sequencing run	1	2.053	2.0526	5.4676	*0.02201*	*0.0010*
Sample type	1	7.733	7.7328	20.5986	*0.08294*	*0.0010*
Study group	1	0.401	0.4008	1.0677	0.0043	0.3420
Sex	1	0.44	0.4402	1.1727	0.00472	0.2740
Age (10 yrs)	5	1.148	0.2296	0.6116	0.01231	0.9920
Residuals	217	81.463	0.3754	-	0.87372	-
Total	226	93.237	-	-	1	-
**Sequencing run 1 and 3**						
**Variables**	**Df**	**Sum of squares**	**Mean squares**	**F.Model**	**R2**	**Pr(>F)**
Sequencing run	1	0.357	0.3567	1.0252	0.00887	0.3760
Sample type	1	5.373	5.3727	15.4401	*0.13359*	*0.0010*
Study group	1	0.162	0.1619	0.4652	0.00402	0.9240
Sex	1	0.205	0.2046	0.5879	0.00509	0.7890
Age (10 yrs)	5	1.413	0.2826	0.812	0.03513	0.7780
Residuals	94	32.709	0.348	-	0.8133	-
Total	103	40.218	-	-	1	-
**Sequencing run 2 and 3**						
**Variables**	**Df**	**Sum of squares**	**Mean squares**	**F.Model**	**R2**	**Pr(>F)**

Sequencing run	1	0.243	0.2433	0.6235	0.00454	0.7930
Sample type	1	3.616	3.6161	9.2676	*0.06753*	*0.0010*
Study group	1	0.461	0.4605	1.1802	0.0086	0.2410
Sex	1	0.515	0.5153	1.3207	0.00962	0.1850
Age (10 yrs)	3	1.502	0.5006	1.2831	0.02805	0.1390
Residuals	121	47.213	0.3902	-	0.88166	-
Total	128	53.55	-	-	1	-
**Jaccard, pairwise, adjusted PERMANOVA on OW and BAL together**						
**Sequencing run 1 and 2**						
**Variables**	**Df**	**Sum of squares**	**Mean squares**	**F.Model**	**R2**	**Pr(>F)**
Sequencing run	1	3.697	3.6974	9.9414	*0.04183*	*0.0010*
Sample type	1	1.309	1.3086	3.5184	*0.01481*	*0.0010*
Study group	1	0.536	0.5364	1.4422	0.00607	0.0960
Sex	1	0.467	0.4674	1.2566	0.00529	0.1850
Age (10 yrs)	5	1.666	0.3331	0.8957	0.01885	0.7500
Residuals	217	80.706	0.3719	-	0.91316	-
Total	226	88.381	-	-	1	-
**Sequencing run 1 and 3**						
**Variables**	**Df**	**Sum of squares**	**Mean squares**	**F.Model**	**R2**	**Pr(>F)**
Sequencing run	1	0.795	0.79466	2.3057	*0.02175*	*0.0050*
Sample type	1	1.119	1.11911	3.2471	*0.03064*	*0.0020*
Study group	1	0.203	0.20334	0.59	0.00557	0.9060
Sex	1	0.466	0.46559	1.3509	0.01275	0.1730
Age (10 yrs)	5	1.55	0.30992	0.8992	0.04242	0.6910
Residuals	94	32.397	0.34465	-	0.88688	-
Total	103	36.529	-	-	1	-
**Sequencing run 2 and 3**						
**Variables**	**Df**	**Sum of squares**	**Mean squares**	**F.Model**	**R2**	**Pr(>F)**
Sequencing run	1	0.591	0.59076	1.5175	0.01157	0.0610
Sample type	1	0.711	0.71073	1.8257	*0.01392*	*0.0360*
Study group	1	0.774	0.77426	1.9889	*0.01516*	*0.0140*
Sex	1	0.492	0.49191	1.2636	0.00963	0.1540
Age (10 yrs)	3	1.389	0.46293	1.1892	0.0272	0.1340
Residuals	121	47.104	0.38929	-	0.92251	-
Total	128	51.06	-	-	1	-

OW: oral wash, BAL: bronchoalveolar lavage, yrs: years. Differences in beta diversity were tested with permuted analysis of variance (PERMANOVA) adjusted for sample type, study group, sex, and age (permutations = 10000).
