# Supplementary material for: The pulmonary mycobiome—A study of subjects with and without chronic obstructive pulmonary disease
Source: PLoS One. 2021 Apr 7;16(4):e0248967. doi: 10.1371/journal.pone.0248967 (PMC8026037; doi:10.1371/journal.pone.0248967)
Supplement: S6 Table — Comparisons were done (A) merged and (B) pairwise. OW: oral wash, BAL: bronchoalveolar lavage. (PDF) [file pone.0248967.s013.pdf]

The pulmonary mycobiome - a study of subjects with and without chronic obstructive pulmonary disease

Supporting Information, S6 Table

Einar M. H. Martinsen[1*], Tomas M. L. Eagan[1,2], Elise O. Leiten[1], Ingvild Haaland[1], Gunnar R. Husebø[1,2], Kristel S. Knudsen[2], Christine Drengenes[1,2], Walter Sanseverino[3], Andreu Paytuví-Gallart[3], and Rune Nielsen[1,2]

[1]Department of Clinical Science, University of Bergen, Bergen, Norway

[2]Department of Thoracic Medicine, Haukeland University Hospital, Bergen, Norway

[3]Sequentia Biotech SL, Barcelona, Spain

* Corresponding author

E-mail: einar.martinsen@uib.no

S6 Table. Permuted multivariate analysis of beta-dispersion using Bray-Curtis and Jaccard distances. Comparisons were done (A) merged and (B) pairwise.

(A)

Bray-Curtis, Permuted multivariate analysis of beta-dispersion on OW and BAL together						
**Variables**	**Df**	**Sum of squares**	**Mean squares**	**F**	**N.Perm**	**Pr(>F)**
Sequencing run	2	0.0283	0.014125	0.4192	1000	0.6913
Residuals	227	7.6481	0.033692	-	-	-
**Jaccard, Permuted multivariate analysis of beta-dispersion on OW and BAL together**						
**Variables**	**Df**	**Sum of squares**	**Mean squares**	**F**	**N.Perm**	**Pr(>F)**
Sequencing run	2	0.13686	0.068429	6.4239	1000	*0.001998*
Residuals	227	2.41808	0.010652	-	-	-

(B)

Bray-Curtis, pairwise Permuted multivariate analysis of beta-dispersion on OW and BAL together. Observed p-value below diagonal, permuted p-value above diagonal			
**Pairs**	First	Second	Third
First	-	0.3976	0.6553
Second	0.41555	-	0.5744
Third	0.74603	0.63992	-
**Jaccard, pairwise Permuted multivariate analysis of beta-dispersion on OW and BAL together. Observed p-value below diagonal, permuted p-value above diagonal**			
**Pairs**	First	Second	Third
First	-	*0.003996*	0.2418
Second	*0.0020067*	-	*0.033*
Third	0.2095527	*0.0278263*	-

OW: oral wash, BAL: bronchoalveolar lavage.
