# Supplementary material for: The pulmonary mycobiome—A study of subjects with and without chronic obstructive pulmonary disease
Source: PLoS One. 2021 Apr 7;16(4):e0248967. doi: 10.1371/journal.pone.0248967 (PMC8026037; doi:10.1371/journal.pone.0248967)
Supplement: S7 Table — ANCOM v2: the second version of analysis of composition of microbiomes, MicrobiomeDDA: Microbiome Differential Distribution Analysis omnibus test, ALDEx2: the second version of ANOVA-Like Differential Expression, OW: oral wash, BAL: bronchoalveolar lavage. The ALDEx2 approach works poorly if there are only a small number of features (less than about 50). The most conservative value in ANCOM v2 has been used in the analyses (i.e. 0.9). (PDF) [file pone.0248967.s014.pdf]

The pulmonary mycobiome - a study of subjects with and without chronic obstructive pulmonary disease

Supporting Information, S7 Table

Einar M. H. Martinsen[1*], Tomas M. L. Eagan[1,2], Elise O. Leiten[1], Ingvild Haaland[1], Gunnar R. Husebø[1,2], Kristel S. Knudsen[2], Christine Drengenes[1,2], Walter Sanseverino[3], Andreu Paytuví-Gallart[3], and Rune Nielsen[1,2]

[1]Department of Clinical Science, University of Bergen, Bergen, Norway

[2]Department of Thoracic Medicine, Haukeland University Hospital, Bergen, Norway

[3]Sequentia Biotech SL, Barcelona, Spain

* Corresponding author

E-mail: einar.martinsen@uib.no

S7 Table. Differential abundance/distribution testing on sequencing run using ANCOM v2, MicrobiomeDDA, and ALDEx2.

**Data**	**ANCOM v2**	**MicrobiomeDDA**	**ALDEx2**
**OW and BAL together**			
All sequencing runs	Candida and Sarocladium	NA. Needs to be two levels	NA. Needs to be two levels
Sequencing run 1 vs 2	Candida and **Sarocladium**	**Sarocladium**, Aspergillus, Ophiostoma, Ascomycota, and Malassezia	**Sarocladium**
Sequencing run 1 vs 3	No significant taxa	Malassezia and Sarocladium	No significant taxa
Sequencing run 2 vs 3	Candida	No significant taxa	Candida
**OW**			
All sequencing runs	Sarocladium	NA. Needs to be two levels	NA. Needs to be two levels
Sequencing run 1 vs 2	**Sarocladium**	**Sarocladium**	**Sarocladium**
Sequencing run 1 vs 3	No significant taxa	No significant taxa	Too few taxa
Sequencing run 2 vs 3	No significant taxa	No significant taxa	No significant taxa
**BAL**			
All sequencing runs	Candida and Sarocladium	NA. Needs to be two levels	NA. Needs to be two levels
Sequencing run 1 vs 2	Candida and **Sarocladium**	**Sarocladium**, Mycosphaerella, Candida, and Penicillium	**Sarocladium**
Sequencing run 1 vs 3	No significant taxa	Mycosphaerella	Too few taxa
Sequencing run 2 vs 3	Candida	No significant taxa	Candida

ANCOM v2: the second version of analysis of composition of microbiomes, MicrobiomeDDA: Microbiome Differential Distribution Analysis omnibus test, ALDEx2: the second version of ANOVA-Like Differential Expression, OW: oral wash, BAL: bronchoalveolar lavage. The ALDEx2 approach works poorly if there are only a small number of features (less than about 50). The most conservative value in ANCOM v2 has been used in the analyses (i.e. 0.9).
