# Supplementary material for: The pulmonary mycobiome—A study of subjects with and without chronic obstructive pulmonary disease
Source: PLoS One. 2021 Apr 7;16(4):e0248967. doi: 10.1371/journal.pone.0248967 (PMC8026037; doi:10.1371/journal.pone.0248967)
Supplement: S1 File — (PDF) [file pone.0248967.s015.pdf]

The pulmonary mycobiome - a study of subjects with and without chronic obstructive pulmonary disease

Supporting Information, S1 File

Einar M. H. Martinsen[1*], Tomas M. L. Eagan[1,2], Elise O. Leiten[1], Ingvild Haaland[1], Gunnar R. Husebø[1,2], Kristel S. Knudsen[2], Christine Drengenes[1,2], Walter Sanseverino[3], Andreu Paytuví-Gallart[3], and Rune Nielsen[1,2]

[1]Department of Clinical Science, University of Bergen, Bergen, Norway

[2]Department of Thoracic Medicine, Haukeland University Hospital, Bergen, Norway

[3]Sequentia Biotech SL, Barcelona, Spain

* Corresponding author

E-mail: einar.martinsen@uib.no

S1 File. Bioinformatic processing.

The bioinformatic processing is shown in Fig 1 in the main manuscript. In total, 579 samples from 193 participants were included, sequenced on three separate runs (Fig 1). Samples from a given participant were always sequenced in the same run. The number of OW, BAL, and negative control samples were identical within each run, and samples from controls and participants with COPD were represented in every run. Four samples were lost due to a lack of overlapping forward and reverse reads. Further five samples were removed by ITSxpress. Merging of sequencing runs post DADA2 resulted in 1250 ASVs, but the number was nearly halved by LULU curation. The number of ASVs dropped considerably after removal of low-abundance ASVs, but the number of sequence reads were less impacted. A quarter of the remaining sequences originated from ASVs presumed to be contamination (listed in S1 Table). We removed negative control samples prior to the analyses. Finally, four BAL samples were removed because of a lacking information whether they were the first or the second BAL fraction.
