# Supplementary material for: The pulmonary mycobiome—A study of subjects with and without chronic obstructive pulmonary disease
Source: PLoS One. 2021 Apr 7;16(4):e0248967. doi: 10.1371/journal.pone.0248967 (PMC8026037; doi:10.1371/journal.pone.0248967)
Supplement: Attachment — Submitted filename: response_to_reviewers_and_editor_Martinsen.docx [file pone.0248967.s016.docx]

Bergen 12^th^ of February 2021

Concerning manuscript PONE-D-20-36443, *The pulmonary mycobiome - a study of subjects with and without chronic obstructive pulmonary disease*.

**Dear Professor Sanjay Haresh Chotirmall**

Thank you for the valuable comments to our paper, and for giving us the possibility to submit a revised version. The original comments of the reviewers and our responses follow. We have added numbering to the comments (C) and our answers (A) to enhance reading and further correspondence.

Editors comments:

C1	Thank you for submitting your manuscript to PLOS ONE. After careful consideration, we feel that it has merit but does not fully meet PLOS ONE’s publication criteria as it currently stands. Therefore, we invite you to submit a revised version of the manuscript that addresses the points raised during the review process.

Please note that there are significant issues raised during the review process focused on the technical aspects of the data acquisition including controls that need to be addressed (please see reviewers comments below). Data availability has to also comply with PLOS policies and our reviewers were not able to access your raw data. Please ensure all of this and he below comments are fully addressed in any revision.

A1	We do believe that we have been able to address all comments from the reviewers, and that these comments have led to improvement of the manuscript. There has been a mistake in the sharing of the DOI for access to our raw data, and the former submission included the final DOI which is inactivated until the paper is accepted. We apologise for the inconvenience this has caused. The temporarily URL for the review process is:

https://datadryad.org/stash/share/1V9eIhBEDmwNdcOqTGfyewO_lcfQlhlyodHDiizTC0U

The URL for the review process given above is now included in the submission as well.

C2	Please ensure that your manuscript meets PLOS ONE's style requirements, including those for file naming. The PLOS ONE style templates can be found at

https://journals.plos.org/plosone/s/file?id=wjVg/PLOSOne_formatting_sample_main_body.pdf and

https://journals.plos.org/plosone/s/file?id=ba62/PLOSOne_formatting_sample_title_aauthor_affiliations.pdf

A2	We have done some formatting changes to the manuscript and file names to meet PLOS ONE´s requirements. Specifically, figure/table legends and citations are altered, and S1 Appendix is replaced by S1 File. We hope that you find the changes satisfactory.

C3	We noted in your submission details that a portion of your manuscript may have been presented or published elsewhere. ("Parts of the content of this manuscript has been presented at the National Microbiota Conference in Norway, and an abstract was published in an online programme. It was also accepted for an oral presentation at the ERS International Congress in 2019, and published as an abstract in the European Respiratory Journal.") Please clarify whether this conference was peer-reviewed and formally published. If this work was previously peer-reviewed and published, in the cover letter please provide the reason that this work does not constitute dual publication and should be included in the current manuscript.

A3	That is correct. The abstract from the presentation at the National Microbiota Conference in Norway can be found in the 2019 program here:

https://microbiota.no/previous-conferences/

The abstract from the ERS International Congress in 2019 can be found here:

https://erj.ersjournals.com/content/54/suppl_63/OA1601

Both abstracts included preliminary results only, and the content was limited compared to the current manuscript. For ERS congress abstracts, authors retain copyright. The presentation abstracts were by no means exhaustive in regards to data, methods, or discussion, which is why we argue that these abstracts do not constitute dual publications.

C4	Thank you for stating the following in the Competing Interests section:

"I have read the journal's policy and the authors of this manuscript have the following competing interests:

EMHM, EOL, IH, GRH, KSK, CD, WS, and APG declare no conflict of interest.

RN reports grants from The endowment of timber merchant A. Delphin and wife (The Norwegian Medical Association) and grants from GlaxoSmithKline during the conduct of the study, and grants and personal fees from AstraZeneca, grants and personal fees from GlaxoSmithKline, grants and personal fees from Boehringer Ingelheim, and grants from Novartis outside the submitted work.

TMLE reports grants from Helse Vest (Western Norway Regional Health Authority) during the conduct of the study, and personal fees from Boehringer Ingelheim outside the submitted work."

Please confirm that this does not alter your adherence to all PLOS ONE policies on sharing data and materials, by including the following statement: "This does not alter our adherence to PLOS ONE policies on sharing data and materials.” (as detailed online in our guide for authors http://journals.plos.org/plosone/s/competing-interests). If there are restrictions on sharing of data and/or materials, please state these. Please note that we cannot proceed with consideration of your article until this information has been declared.

Please include your updated Competing Interests statement in your cover letter; we will change the online submission form on your behalf.

A4	Please see A5.

C5	Thank you for stating the following in the Financial Disclosure section:

"The MicroCOPD study was funded by unrestricted grants and fellowships from Helse Vest, GlaxoSmithKline, Bergen Medical Research Foundation, and the Endowment of Timber Merchant A. Delphin and Wife through the Norwegian Medical Association.

The funders had no role in study design, data collection and analysis, decision to publish, or preparation of the manuscript."

We note that one or more of the authors are employed by a commercial company: 3Sequentia Biotech SL, Carrer D'Àlaba 61, 08005, Barcelona, Spain

(1)Please provide an amended Funding Statement declaring this commercial affiliation, as well as a statement regarding the Role of Funders in your study. If the funding organization did not play a role in the study design, data collection and analysis, decision to publish, or preparation of the manuscript and only provided financial support in the form of authors' salaries and/or research materials, please review your statements relating to the author contributions, and ensure you have specifically and accurately indicated the role(s) that these authors had in your study. You can update author roles in the Author Contributions section of the online submission form.

Please also include the following statement within your amended Funding Statement.

“The funder provided support in the form of salaries for authors [insert relevant initials], but did not have any additional role in the study design, data collection and analysis, decision to publish, or preparation of the manuscript. The specific roles of these authors are articulated in the ‘author contributions’ section.”

If your commercial affiliation did play a role in your study, please state and explain this role within your updated Funding Statement.

Please also provide an updated Competing Interests Statement declaring this commercial affiliation along with any other relevant declarations relating to employment, consultancy, patents, products in development, or marketed products, etc.

Within your Competing Interests Statement, please confirm that this commercial affiliation does not alter your adherence to all PLOS ONE policies on sharing data and materials by including the following statement: "This does not alter our adherence to PLOS ONE policies on sharing data and materials.” (as detailed online in our guide for authors http://journals.plos.org/plosone/s/competing-interests) . If this adherence statement is not accurate and there are restrictions on sharing of data and/or materials, please state these. Please note that we cannot proceed with consideration of your article until this information has been declared.

Please include both an updated Funding Statement and Competing Interests Statement in your cover letter. We will change the online submission form on your behalf.

Please know it is PLOS ONE policy for corresponding authors to declare, on behalf of all authors, all potential competing interests for the purposes of transparency. PLOS defines a competing interest as anything that interferes with, or could reasonably be perceived as interfering with, the full and objective presentation, peer review, editorial decision-making, or publication of research or non-research articles submitted to one of the journals. Competing interests can be financial or non-financial, professional, or personal. Competing interests can arise in relationship to an organization or another person. Please follow this link to our website for more details on competing interests: http://journals.plos.org/plosone/s/competing-interests

A5	Thank you for emphasising this important issue. Our updated Competing Interest statement follows:

"I have read the journal's policy and the authors of this manuscript have the following competing interests:

EMHM, EOL, IH, GRH, KSK, and CD declare no conflict of interest. WS and APG are employed at Sequentia Biotech SL. RN reports grants from The endowment of timber merchant A. Delphin and wife (The Norwegian Medical Association) and grants from GlaxoSmithKline during the conduct of the study, and grants and personal fees from AstraZeneca, grants and personal fees from GlaxoSmithKline, grants and personal fees from Boehringer Ingelheim, and grants from Novartis outside the submitted work. TMLE reports grants from Helse Vest (Western Norway Regional Health Authority) during the conduct of the study, and personal fees from Boehringer Ingelheim outside the submitted work. This does not alter our adherence to PLOS ONE policies on sharing data and materials."

Our updated Funding statement follows:

"The MicroCOPD study was funded by unrestricted grants and fellowships from Helse Vest, GlaxoSmithKline, Bergen Medical Research Foundation, and the Endowment of Timber Merchant A. Delphin and Wife through the Norwegian Medical Association.

The MicroCOPD funders had no role in study design, data collection and analysis, decision to publish, or preparation of the manuscript. Sequentia Biotech SL provided support in the form of salaries for authors [WS and APG], but not study design or data collection. All funding of data collection and laboratory analyses were from the MicroCOPD Study. WS and APG both contributed to interpretation of results, and preparation of the manuscript. The specific roles of all authors are articulated in the ‘author contributions’ section."

Thus, author contributions are left unchanged from the initial submission, as we think this is most accurate. If you think we have misunderstood anything, please let us know.

Reviewer 1:

C6	Martinsen EMH et al; evaluated the oral wash and BAL mycobiome in COPD (n=93) and control subjects (n=100) from a single center in Western Norway. Candida was the dominant genus in all samples with higher abundance in oral compared to BAL samples. No difference in alpha and beta-diversity between study groups and ICS used.

This is the first study using BAL to access the airway mycobiome in COPD, however there were numerous weakness which need to be address

The control subjects were defined as no COPD and other lung diseases, however 22 of the control subjects had FEV/FVC <0.7, is this group of patient smoker? why are their lung function obstructive? Any underlying bronchiectasis or asthma which explained the abnormal lung function? As their spirometry is abnormal is hard to believe that these individuals have no underlying lung diseases, perhaps they should be removed from the control group and reanalyzed.

A6	We would like to thank the reviewer for the comment. In the current study, COPD was defined as chronic airway obstruction (low FEV_1_/FVC) *in presence of respiratory symptoms*. This adheres to the GOLD guidelines, in which following information on the establishment of a COPD diagnosis is found:

“… the presence of a post-bronchodilator FEV1/FVC < 0.70 confirms the presence of persistent airflow limitation and thus of COPD in patients with appropriate symptoms and significant exposures to noxious stimuli” (1)

Consequently, it is justified that more than an obstructive spirometry is needed to establish a COPD diagnosis. In some instances, the differentiation between COPD and asthma can be challenging, and also differentiation between COPD stage I and healthy smoking controls. After data collection on all study participant was completed, diagnoses where the study physician upon inclusion had marked "questionable", were re-evaluated by three pulmonologists from our research group based on spirometry, radiologic imaging, respiratory symptoms, and disease history. These pulmonologists had long experience with treatment and handling of individuals with obstructive lung diseases. A few controls had a ratio of FEV_1_/FVC lower than 0.7, but did not have symptoms of COPD and remained classified as controls. Furthermore, we specifically asked all participants for other lung and airway diseases.

We agree with the reviewer that it should be interesting to examine the obstructive controls in more detail. The following table includes the obstructive controls and the COPD group:

| **Variable** | **Obstructive controls, n=22** | **COPD, n=93** | **Comparison** |
| --- | --- | --- | --- |
| **Age, mean years (SD)** | 68.9 (7.1) | 67.4 (7.6) | p-value = 0.4047 |
| **FEV_1_, mean % of predicted (SD)** | 97.4 (13.1) | 61.1 (17.3) | *p-value < 0.0001* |
| **Pack years, mean (SD)** | 28.5 (25.8) | 30.0 (17.9) | p-value = 0.7421 |
| **Smoking status (%)** |  |  |  |
| Daily | 13 (59.1) | 22 (23.7) |  |
| Ex-smokers | 6 (27.3) | 70 (75.3) | *p-value < 0.01* |
| Never | 3 (13.6) | 1 (1.1) |  |

Age, FEV_1_ in percentage of predicted, and pack years was tested with a t-test.

Smoking status was tested with Fisher’s exact test. We would like to emphasise that the obstructive controls have significantly higher FEV_1_ values than participants with COPD. The obstructive controls have a pretty high smoking burden, but less than participants with COPD if we merge daily and ex-smokers. Albeit non-significant, obstructive controls are also older, and we know that airflow limitation could be a result of advancing age (2, 3). We believe that a thorough individual examination in our current study prevented overdiagnosis of COPD.

C7	The author mention that PBS was obtained as negative control samples, and contamination removed with Decontam, but it seems that negative control samples are also dominated by Candida (Figure S2), what was the abundance of Candida in the negative control samples?

A7	We made the following rank abundance plot to show distribution of taxa in the different sample types:

OW: oral wash, BAL: bronchoalveolar lavage, NCS: negative control sample. Contaminants identified by Decontam are excluded in OW and BAL, but not NCS.

The mean *Candida* proportions in OW, BAL, and NCS were 0.75, 0.41, and 0.41, respectively, and ranged from 0-1.0 in every sample type. Visually, sample types seem quite similar, but we have found differences in abundance. ANCOM v2 reported that *Candida* and *Sarocladium* were different in abundance between OW and NCS, and BAL and NCS. *Candida* was also reported to differ between OW and NCS by ALDEx2 (see table below):

| **Data** | **ANCOM v2** | **ALDEx2** |
| --- | --- | --- |
| OW vs NCS | **Candida** and Sarocladium | **Candida** |
| BAL vs NCS | Candida and Sarocladium | No significant taxa |

The most conservative value in ANCOM v2 has been used in the analyses (i.e. 0.9).

We would also like to emphasise that differences were seen in DNA yields and read counts between NCS and OW/BAL (please see A14 and A15). By looking at relative abundances (as above), such differences are overseen.

C8	What is the clinical relevance and impact of this study finding?

A8	There are few direct clinical implications of our study, but the ubiquity of fungi shown in our paper highlights that these organisms and their pathogenic potential should be considered, particularly taking into account that the prevalence and severity of mycosis have increased the recent years (i.e. opportunistic infections in immunocompromised individuals) (4). Fungal respiratory infections are often difficult to identify as well as treat, and the widespread use of antibiotics might have facilitated respiratory mycosis resistant to antifungal drugs by influencing the balance between bacterial and fungal infections (5). We have shown in our paper that *Candida*, one of the most well-known fungal pathogens (5), resides in the lungs of healthy people. Colonising *Candida* can cause local or systemic infections through morphological alterations, for instance as a result of changes in the microenvironment or host innate immunity (6). This has been discussed for the gut mycobiome (6), and that similar mechanisms could happen in the lungs are not unlikely. However, our results are limited to genus level. Although *Candida albicans* is the main pathogen causing candidiasis, non‐albicans *Candida* species are becoming increasingly reported as the causative agent (7). To examine clinical implications in detail, more information on species level would be required.

Mycobiomes have also been associated with other inflammatory diseases such as inflammatory bowel diseases (4), justifying investigation of the COPD mycobiome. Previous studies on the COPD mycobiome are limited by low sample sizes and possible contamination. We believe our study provides a reliable characterisation of the COPD mycobiome, which is a prerequisite for further investigations. Based on our observations, we also suggest that ICS use do not significantly affect the lung mycobiome, and therefore has no harmful effect on the lung mycobiome.

We have included more information on the clinical implications in our manuscript (lines 326-330 in file without track changes).

C9	Was the sample collected at stable COPD? how many weeks post exacerbations were patients allow to be recruited for the study?

A9	To investigate the stable state of the microbiome, we postponed participation for subjects that were currently on, or had recently had, a course of systemic antimicrobial treatment or systemic steroid treatment. Specifically, no participant should have used antibiotics last 2 weeks prior to participation, and COPD patients should not have been admitted to hospital due to COPD last 2 weeks. Furthermore, participants with symptoms of an ongoing systemic or respiratory infection could not attend, but had to postpone participation. A modification of Anthonisen´s symptom criteria (8) were used to rule out or confirm a likely respiratory infection or acute exacerbation of COPD, where presence of two major symptoms (i.e. increased dyspnoea, colour change or increased sputum) or one major and one minor symptom (increased cough, sore throat, runny/stuffed nose, asthenia, wheezing from the chest) excluded attendance. We have included the information in the manuscript (line 112-115 in file without track changes).

C10	Is there any patient on long term oral corticosteroid, antifungal or antibiotics?

A10	No participant should have used antibiotics last 2 weeks prior to participation. Three participants with COPD had oral corticosteroids recorded on their medication list at the time of participation. Two of these three participants on oral corticosteroids used oral corticosteroids when needed, and one of them had her/his last course of cortisone/prednisolone 13 months ago, while the other couldn´t remember or thought she/he hadn´t had any course of cortisone/prednisolone. The last of the three participants was on a course of prednisolone 10 mg 1x1 at the time of participation due to back pain. The course had been initiated 9 days before participation, and she/he had taken the last dose 3 hours and 10 minutes before bronchoscopy start. We assess any potential impact on the results from this participant to be small considering the sample size of the study.

C11	What is the breakdown of patient in each group (Figure 4) how many patients in COPD grade I/II and COPD grade III/IV?

A11	Number of samples in each group was as follows:

Figure 4A (unpaired): Control BAL: 40, control OW: 76, COPD grade I/II BAL: 29, COPD grade I/II OW: 50, COPD grade III/IV OW: 23, COPD grade III/IV BAL: 12.

Figure 4B (paired): Control BAL: 31, control OW: 31, COPD grade I/II BAL: 24, COPD grade I/II OW: 24, COPD grade III/IV OW: 9, COPD grade III/IV BAL: 9.

Information on number of samples is now included in the figure legend for Fig 4.

C12	Please ensure sequencing data is publicly available

A12	We apologise, this has been corrected. Please see A1.

C13	The weakness including cross-sectional nature, interaction between various kingdom and host responses were not access in this study and should be discuss

A13	We would like to thank the reviewer for the comments. We agree that another study design and inclusion of analyses on interactions between fungi and other organisms and the host could have added value to our current analyses, and further studies should certainly consider this. We have now addressed this as a limitation in our discussion.

Reviewer 2:

C14	What DNA yields were achieved in samples (OW and BAL) vs negative extraction blanks? This is a critical point. This data should be plotted and included in the manuscript. Both the DNA and fungal yields need to be quantified in some way to demonstrate that the sampling technique actually works i.e. a significantly higher DNA/fungal/Fungal DNA yield is obtained in samples relative to background contamination. Any samples where this cannot be safely concluded should probably be excluded.

A14	We appreciate the suggested revision. Unfortunately, we did not perform any qPCR in our study, but we have quantified DNA yields prior to sequencing using Qubit. We suggest including a new figure in the Supplementary Information, S5 Fig, showing distributions of DNA yields in the different sample types. The figure is included here for simplicity:

BAL: bronchoalveolar lavage, NCS: Negative control sample, OW: oral wash.

There were statistically significant differences between all sample types tested with Wilcoxon signed-rank test as a paired test (p < 0.001 for all tests).

Mean Qubit concentrations: BAL = 13.0, NCS = 0.2, OW = 17.5.

Median Qubit concentrations: BAL = 7.8, NCS = 0.1, OW =13.1.

Interquartile ranges Qubit concentrations: BAL = 11.3, NCS = 0.1, OW = 19.9.

We think this indicate that OW and BAL samples from the current study are reliable and not the result of background contamination.

C15	What was the total raw sequencing depth and total read count in samples (OW and BAL) vs negative extraction blanks (NCS)? Please include a figure/table. Figure S2 shows only relative abundance i.e. the stacked bar charts are proportionally scaled and there is no way to determine how many reads were detected in the NCS compared to samples. A single figure representing aggregate read numbers for each taxonomic classification (genus level) for OW NCS and BAL would be useful. The multiple graphs in figure S2 are informative but a little difficult to digest at first glance. An accompanying graph summarising the aggregate comparison would help – i.e. Total read counts for NCS, OW and BAL for all reported OTUs.

A15	Please see S4 Table in the Supplementary Information. The S4 Table is a summary of the total and mean read count at various stages of the study. The table is included here for simplicity:

| **Data** | **Number of samples** | **Read/sequence count** | **Mean read/sequence count** |
| --- | --- | --- | --- |
| **Raw data** | | | |
| NCS | 189 | 667,033 | 3,529 |
| OW | 193 | 22,933,493 | 118,826 |
| BAL | 193 | 20,348,685 | 105,434 |
| **Post ITSxpress** | | | |
| NCS | 184 | 529,519 | 2,878 |
| OW | 193 | 11,907,308 | 61,696 |
| BAL | 193 | 5,790,865 | 30,004 |
| **Post DADA2** | | | |
| NCS | 161 | 472,574 | 2,935 |
| OW | 193 | 11,236,458 | 58,220 |
| BAL | 193 | 5,317,673 | 27,553 |
| **Post LULU** | | | |
| NCS | 157 | 410,205 | 2,613 |
| OW | 193 | 10,881,332 | 56,380 |
| BAL | 192 | 4,300,589 | 22,399 |
| **Without low abundance**  **and contaminants** | | | |
| NCS | 122 | 73,658 | 604 |
| OW | 192 | 9,490,666 | 49,431 |
| BAL | 185 | 1,663,488 | 8,992 |

NCS: Negative control sample, OW: oral wash, BAL: bronchoalveolar lavage, DADA2: Divisive Amplicon Denoising Algorithm version 2.

It is possible to read from the table that the read count is lower in NCS compared to OW and BAL.

C16	For many subjects, negative controls (NCS) are highly similar to the samples (BAL/OW). Unless there is some other evidence for a signal in these samples (i.e. much higher DNA yield/read count in the samples vs NCS) then these must be excluded from analysis. If NCS profiles match either a BAL or OW sample and have comparable yield in terms of DNA yield or total number of classified reads then there is no way to tell whether this signal is coming from the sample or represents stochastic background contamination signal. Such cases should be systematically identified, excluded and the analysis repeated. This could impact interpretation regarding clinical association.

A16	We appreciate the emphasis on this important issue. Contaminants have been removed by using the Decontam algorithm*.* And we have previously shown that both the DNA yield and the read counts are significantly different for OW and BAL compared to NCS (please see A14 and A15). We believe that removing samples that resemble the NCS is problematic, because it might be that the true constituents of the environmental microbiota, that influence the NCS, are the same as we find in most lung samples.

C17	Data and code repository not accessible (https://doi.org/10.5061/dryad.w3r2280nz)

A17	We apologise, this has been corrected. Please see A1.

C18	Batch effects. The authors assessed batch effects associated with sequencing runs. Assuming the DNA extractions were not all done as a single batch, the same analysis should be performed for DNA extraction batch effects (between-batch effects).

A18	We have repeated the analysis focusing on the different DNA extraction kit lot numbers that were used. Unfortunately, we missed information on extraction kit used for 114 of 230 samples included for diversity analysis after rarefaction. However, with the available information we had, we created PCoA plots using Bray-Curtis (upper plots) and Jaccard (lower plots) for (A) processed OW and BAL samples, (B) OW samples only, (C) BAL samples only, and (D) processed OW and BAL samples, together with NCS processed with ITSxpress, DADA2, and LULU, but not Decontam. The PCoA plots are inserted below:

PCoA plots shown here are made with Bray-Curtis.

PCoA plots shown here are made with Jaccard.

Information on missing samples were included in the PCoA plots. In some of the plots, it looks like samples with missing information on lot numbers cluster separately from the rest. However, inside the missing group, several different lot numbers could have been used. Unfortunately, we do not know that. The rest of the samples seem to us randomly scattered in the plots.

We also tested differences in beta diversity with PERMANOVA. This was only possible if missing data was removed. We calculated dispersion (variances) within each lot number group prior to the PERMANOVA analysis. Variances differed by groups for OW and BAL samples analysed with Jaccard, OW samples analysed with Jaccard, all BAL samples, and OW, BAL, and NCS samples analysed with Jaccard. PERMANOVA were thus precluded in these samples. PERMANOVA analyses for the remaining samples are shown below:

OW and BAL, Bray-Curtis

| **Variables** | **Df** | **SumsOfSqs** | **MeanSqs** | **F.Model** | **R2** | **Pr(>F)** |
| --- | --- | --- | --- | --- | --- | --- |
| Extraction lot number | 4 | 1.939 | 0.48482 | 1.2318 | 0.04045 | 0.1527 |
| Sample type | 1 | 2.708 | 2.7077 | 6.8797 | 0.05648 | 1.00E-04 |
| Residuals | 110 | 43.294 | 0.39358 |  | 0.90307 |  |
| Total | 115 | 47.941 |  |  | 1 |  |

OW only, Bray-Curtis

| **Variables** | **Df** | **SumsOfSqs** | **MeanSqs** | **F.Model** | **R2** | **Pr(>F)** |
| --- | --- | --- | --- | --- | --- | --- |
| Extraction lot number | 4 | 0.8881 | 0.22203 | 0.61498 | 0.03349 | 0.9165 |
| Residuals | 71 | 25.633 | 0.36103 |  | 0.96651 |  |
| Total | 75 | 26.5211 |  |  | 1 |  |

OW, BAL, and NCS, Bray-Curtis

| **Variables** | **Df** | **SumsOfSqs** | **MeanSqs** | **F.Model** | **R2** | **Pr(>F)** |
| --- | --- | --- | --- | --- | --- | --- |
| Extraction lot number | 4 | 2.465 | 0.6163 | 1.5647 | 0.03972 | 0.012 |
| Sample type | 2 | 6.419 | 3.2096 | 8.1487 | 0.10344 | 1.00E-04 |
| Residuals | 135 | 53.174 | 0.3939 |  | 0.85684 |  |
| Total | 141 | 62.058 |  |  | 1 |  |

In samples tested with PERMANOVA, significant effects from lot numbers were only observed when NCS were included. We believe most emphasis should be put on the plots and analyses without several sample types, as it is hard to adjust for the differences between sample types. Indeed, the p-value for sample type from the PERMANOVA with NCS is very small, and R2 (i.e. variation in distances explained by the particular variable) is 2.6 fold higher than the extraction lot numbers.

Based on the PCoA plots and the PERMANOVA we do not believe batch effects were introduced from the different DNA extraction lots.

C19	Confusing statement in the discussion concerning “medians were dominated by zeros, comparable to the 0.03 found in the CF study”. Please revise and state clearly what is meant by this without requiring the reader to refer to another paper.

A19	We realise that this was confusing, and have tried to clarify in the manuscript.

C20	Final paragraph; “still in its infancy” > “still in their infancy”

A20	Thank you for making us aware of this misspelling. We have changed the sentence in the manuscript as suggested.

While revising the draft, some minor changes were made:

- A minor error reporting in age in the demography table was noticed. The mean age for participants with COPD was changed from 67.4 to 67.5, and the standard deviation (SD) for age in controls was changed from 8.4 to 8.5. Pack years for controls were 16.6 with SD 14.3, not 17.6 and SD 17.5. We consider these changes to not impact other parts of the manuscript.

- Changed from “fourfold” to “threefold” on line 69 in file without track changes at the end of the introduction.

- Included full name of the ethics committee (line 103 in file without track changes).

- Corrected misspelling on line 281 in file without track changes by changing “and between were” to “was”.

- Added “*k__Fungi*” in the Fig 3 figure legend.

- Added “Gagnat” to Ane´s name in Acknowledgement.

We hope that you find the revision satisfactory, but are of course happy to provide a second revision if necessary. All changes are marked with “track changes” function in the revised manuscript.

We thank the reviewers and the editor for helping improve our paper. Please do not hesitate to contact the corresponding author if unresolved issues remain.

On behalf of the writing group:

Einar Marius Hjellestad Martinsen

Department of clinical science, University of Bergen

[einar.martinsen@uib.no](mailto:einar.martinsen@uib.no)

**References in response to reviewers**

1.	Global Initiative for Chronic Obstructive Lung Disease. Global Strategy for the Diagnosis, Management, and Prevention of Chronic Obstructive Pulmonary Disease, 2020. Available from: https://goldcopd.org/.

2.	Mannino DM, Sonia Buist A, Vollmer WM. Chronic obstructive pulmonary disease in the older adult: what defines abnormal lung function? Thorax. 2007;62(3):237-41.

3.	Price DB, Yawn BP, Jones RC. Improving the differential diagnosis of chronic obstructive pulmonary disease in primary care. Mayo Clin Proc. 2010;85(12):1122-9.

4.	Cui L, Morris A, Ghedin E. The human mycobiome in health and disease. Genome Med. 2013;5(7):63.

5.	Nguyen LD, Viscogliosi E, Delhaes L. The lung mycobiome: an emerging field of the human respiratory microbiome. Front Microbiol. 2015;6:89.

6.	Lagunes L, Rello J. Invasive candidiasis: from mycobiome to infection, therapy, and prevention. Eur J Clin Microbiol Infect Dis. 2016;35(8):1221-6.

7.	Sanguinetti M, Posteraro B, Lass-Flörl C. Antifungal drug resistance among Candida species: mechanisms and clinical impact. Mycoses. 2015;58 Suppl 2:2-13.

8.	Anthonisen NR, Manfreda J, Warren CP, Hershfield ES, Harding GK, Nelson NA. Antibiotic therapy in exacerbations of chronic obstructive pulmonary disease. Ann Intern Med. 1987;106(2):196-204.
